# Supplementary material for: Potential SARS-CoV-2 infectiousness among asymptomatic healthcare workers
Source: PLoS One. 2021 Dec 17;16(12):e0260453. doi: 10.1371/journal.pone.0260453 (PMC8682911; doi:10.1371/journal.pone.0260453)
Supplement: S1 File — (PDF) [file pone.0260453.s001.pdf]

KAROLINSKA
UNIVERSITETSLABORATORIET

Följebrev
2020-03-30 Dnr:

Karolinska Universitetslaboratoriet
Joakim Dillner
Telefon: 072-4682460
Joakim.Dillner@sll.se

Etikprövningsmyndigheten
Box 2110
750 02 Uppsala

Studier av pågående och genomgången SARS-CoV-2 infektion (som orsakar COVID-19) på akutsjukhus i Stockholms län

Etikansökan avser en studie att undersöka om personal och patienter vid akutsjukhus i Stockholm har pågående eller genomgången SARS-CoV-2 infektion. Då antalet smittade av SARS-CoV-2 just nu ökar exponentiellt i Sverige och mängden patienter som behöver vård för COVID-19 snabbt ökar, är det av största vikt att snabbt förstå hur infektionen sprids i sjukvården för att kunna planera en god och säker vård.

Om åtgärden visar sig framgångsrik kan smittspridning av SARS-CoV-2 viruset reduceras/förhalas, i synnerhet för den riskgrupp som inlagda på akutsjukhusen utgör, vilket i sin tur kan ha betydande effekter på samhället, på den pågående pandemin samt för vårdens kapacitet och möjlighet att erbjuda fullgod vård.

Studien kan starta omedelbart efter att etiktillstånd erhållits och infrastrukturen för provtagning och provanalys byggts upp. Förväntad start är 2020-04-06. Då provtagningspersonal, laboratorieanalyspersonal, provtagnings- och analysutrustning samt forskningspersoner finns på plats är förutsättningarna för att snabbt kunna genomföra studien goda.

Stockholm 2020-03-30

Med vänlig hälsning

Joakim Dillner, Forskningschef

Karolinska Universitetslaboratoriet

Ansökan om etikprövning
Allmän information och underskrifter

1. Allmän information och underskrifter

1.1. Titel på forskningsprojektet

Ange en beskrivande titel på svenska. Titeln ska gå att förstå för en lekman.

Studier av pågående och genomgången SARS-CoV-2 infektion (som orsakar COVID-19) på akutsjukhus i Stockholms län.

AVGIFTSKATEGORI

1.2. Hur många forskningshuvudmän kommer att ingå i forskningsprojektet?

Forskningshuvudman: Den statliga myndighet eller fysiska eller juridiska person i vars verksamhet forskningen utförs. En fysisk person kan endast undantagsvis vara forskningshuvudman.

☒ En
☐ Flera

Om en: Avgiften för ansökan är 5000 kronor.

1.2.1. [Om Flera] Har samtliga forskningspersoner ett omedelbart samband med endast en av forskningshuvudmännen?

Forskningsperson: De levande människor som forskningen avser.

Omedelbart samband: Forskningspersoner inkluderas i forskningen hos endast en av forskningshuvudmännen.

☒ Ja
☐ Nej

Om Ja: Avgiften för ansökan är 5 000 kronor.

Om Nej: Avgiften för ansökan är 16 000 kronor.

1.3. Avser forskningen klinisk läkemedelsprövning?

Klinisk läkemedelsprövning: Klinisk undersökning på människor av ett läkemedels egenskaper.

☐ Ja
☒ Nej

Om Ja: Avgiften för ansökan är 16 000 kronor oavsett om en eller fler huvudmän deltar i projektet.

1.3.1. [Om Ja 1.3] Ange EudraCT-nummer

YYYY-NNNNNN-CC

1.4. Ska endast befintliga personuppgifter behandlas i projektet?

Ett JA-svar innebär att projektet endast kommer att behandla (exempelvis bearbeta, sammanställa eller samköra) personuppgifter som redan finns i olika register. Det vill säga inga nya personuppgifter kommer att samlas in för att genomföra forskningen.

☐ Ja
☒ Nej

Om Ja: Avgiften för ansökan är 5 000 kronor oavsett om en eller flera huvudmän deltar i projektet.

1.5. Önskas ett rådgivande yttrande?

Om forskningen i ansökan bedöms vara av det slag som inte kräver godkännande av Etikprövningsmyndigheten kan myndigheten ge ett rådgivande yttrande. I ett rådgivande yttrande kan Etikprövningsmyndigheten till exempel meddela att man inte ser några etiska hinder för projektets genomförande.

☒ Ja
☐ Nej

Ansökan om etikprövning

Följebrev vid komplettering av ansökan om etikprövning

1. **Besvara de frågor eller synpunkter som Etikprövningsmyndigheten angett i sitt beslut.**

Av följebrevet ska det framgå hur du ställer dig till myndighetens synpunkter på forskningsprojektet och ansökan. Om du reviderar din ansökan ska det av följebrevet tydligt framgå vilka avsnitt i ansökan du har reviderat och på vilket sätt. Om du även reviderar bilagor ska det framgå vilka bilagor du har reviderat och på vilket sätt. Ansökan och bilagorna ska bifogas med spårade ändringar om de har reviderats. Vid omfattande bilagor ska det även anges på vilka sidor ändringar är gjorda.

Reviderad forskningspersonsinformation har formulerats som särskilt vänder sig till de personer som tillägget avser dvs omvårdnadspersonal (bilaga A) och brukare (bilaga B). Forskninsgpersonsinformationen som vänder sig till akutsjukhusen har uppdaterats efter i) juristgranskning ii) att vi tyvärr ej kunde använda 1177, men använder en motsvarande utsvarstjänst sant iii) att det visat sig att svalgprov är lika bra som näsprov, varför ordet "näsprov" bytts ut mot "svalgprov" så även denna bifogas (Bilaga C).
Ett exklusionskriterium för studien är de som inte lämnat informerat samtycke. De forskningspersoner som inte kan förstå och ge ett välinformerat samtycke exkluderas därför ur studien. Vi kommer i information till medicinskt ansvarig som enrollerar understryka att enrollering inte får ske om forskningspersonen i någon form ger uttryck för att inte vilja delta eller om en ställföreträdare eller anhörig motsätter sig deltagande

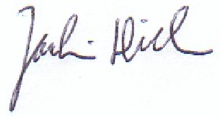

____

Underskrift ansvarig forskare
Joakim Dillner, professor
2020-04-23

ANSVARIGA OCH MEDVERKANDE FORSKARE OCH FORSKNINGSHUVUDMÄN

1.6. Ansvarig huvudman för forskningen (forskningshuvudman)

Forskningshuvudman: Den statliga myndighet eller fysiska eller juridiska person i vars verksamhet forskningen utförs. En fysisk person kan endast undantagsvis vara forskningshuvudman.

Karolinska Universitetssjukhuset

1.7. Behörig företrädare för forskningshuvudmannen

Björn Zoega, Sjukhusdirektör

1.8. Övriga forskningshuvudmän som deltar i projektet:

Som deltagande forskningshuvudman räknas den huvudman inom vars verksamhet forskning kommer att bedrivas, det vill säga där forskningspersoner kommer att inkluderas och/eller där data kommer att behandlas och bearbetas.

Vi planerar att erbjuda andra akutsjukhus inom länet att enrollera patienter till projektet. I dagsläget finns dock bara en huvudman. Om fler tillkommer gör vi en tilläggsansökan.

1.9. Huvudansvarig forskare för projektet (kontaktperson):

Joakim Dillner

1.10. Är den huvudansvariga forskaren disputerad?

Huvudregeln är att den ansvariga forskaren ska vara disputerad inom relevant ämnesområde alternativt inneha motsvarande kompetens. Den som är huvudansvarig forskare ansvarar för att andra medverkande som ska genomföra projektet har tillräcklig kompetens (vetenskaplig och klinisk). Vid läkemedelsprövning ska huvudansvarig forskare dessutom ha tillräcklig kunskap om "Good Clinical Practice" (GCP).

I undantagsfall kan icke disputerad forskare godtas om annan medverkande disputerad forskare uttalat att forskningen sker under aktivt överinseende av denne. Uttalandet ska vara skriftligt och CV för den disputerade ska bifogas.

I den mån sökanden begär att forskningen ska få genomföras utan att en disputerad forskare medverkar får en bedömning ske av samtliga omständigheter i det enskilda fallet. Kompetensen ska bedömas i relation till den forskning som är aktuell och till de etiska frågeställningar som forskningen kan antas ge upphov till. Krav bör kunna ställas på att personen i fråga behärskar aktuella vetenskapliga metoder och i övrigt har visat sig lämpad att hantera ett sådant ansvar. Forskaren i fråga ska också ha erfarenhet av att ta ställning till forskningsetiska problem (se prop. 2002/03:50 s. 100).

Ja

1.10.1. [Om Nej 1.10] **Ange namnet på den [disputerade] forskare som kommer utöva att aktivt överinseende över forskningen.**

Ett skriftligt uttalande om aktivt överinseende samt CV för den som utövar aktivt överinseende ska bifogas ansökan.

Namn Efternamn
Titel
E-postadress

1.11. Andra medverkande forskare:

Ange namn, titel och funktion på de forskare som ska medverka i projektet.

Operativ Direktör Kalle Conneryd Lundgren, Karolinska Universitetssjukhuset
Professor Lars Engstrand, Karolinska Universitetslaboratoriet, Karolinska Universitetssjukhuset
Professor Lars I Eriksson, FoU-chef och överläkare vid Funktion Perioperativ Medicin och Intensivvård, Karolinska Universitetssjukhuset.

ÖVRIG INFORMATION

1.12. Avser ansökan forskning som inbegriper äggdonation?

☐ Ja

☒ Nej

1.13. Avser ansökan forskning med läkemedel för genterapi eller somatisk cellterapi eller läkemedel som innehåller genetiskt modifierade organismer?

☐ Ja

☒ Nej

1.14. Avser ansökan forskning med xenogen cellterapi?

☐ Ja

☒ Nej

1.15. Kommer joniserad strålning ingå i forskningsprojektet?

Med joniserad strålning avses partikelstrålning eller elektromagnetisk strålning som har tillräcklig energi för att jonisera materia. Om joniserad strålning ska ingå i projektet ska ifylld stråldosbilaga bifogas ansökan.

☐ Ja

☒ Nej

BILAGEFÖRTECKNING

1.16. Ange vilka bilagor som medföljer ansökan.

	Bilage-nummer	Namn bilaga	Ledtext
☒	1	Ansökan om etikprövning – Beskrivning av forskningsprojektet	*Formuläret Ansökan om etikprövning- beskrivning av forskningsprojektet är obligatoriskt och ska alltid bifogas ansökan*
☒	2	Ansökan om etikprövning- Beskrivning av biologiskt material	*Om biologiskt material ska nyinsamlas eller om befintligt biologiskt material ska användas i forskningsprojektet är formuläret Ansökan om etikprövning-- Beskrivning av biologiskt material obligatoriskt och ska alltid bifogas ansökan.*
☒	3	Forskningsplan avsedd för fackmän	*Den sammanfattande beskrivningen av forskningsprojektet ska förstås av Etikprövningsmyndighetens samtliga ledamöter. Den kan lämpligen utformas enligt följande:*<br>*Vetenskaplig frågeställning: En redogörelse för det övergripande syftet med det föreslagna forskningsprojektet samt specifika mål (primära och sekundära frågeställningar).*<br>*Områdesöversikt: Ge ett sammandrag av egna och andras forskning och tidigare resultat inom forskningsområdet. Översikten ska tydliggöra det aktuella projektets relevans. Nyckelreferenser ska anges.*<br>*Projektbeskrivning: Gör en sammanfattning av projektets/motsvarande uppläggning. Urval av forskningspersoner, procedurer, metoder med mera ska tydligt redovisas. Det ska framgå hur metoder, urval och procedurer kan ge svar på de specifika frågeställningarna. Om flera delprojekt avses anges sekvens för genomförande och på vilket sätt ett efterföljande delprojekts uppläggning kan bero av resultaten av ett föregående.*<br>*Betydelse: Ge en kortfattad redogörelse för projektets betydelse för forskningsområdet.*<br>*Preliminära resultat: Kan i förekommande fall anges.*
☐	4	Annonsmaterial för rekrytering av forskningspersoner	*Etikprövningsmyndigheten behöver alltid ta del av allt annonsmaterial som ska användas vid rekryteringen. Materialet ska vara skrivet på svenska*
☒	5	Information som kommer att ges till forskningspersonerna i samband med tillfrågan om deltagande	*Etikprövningsmyndigheten behöver alltid ta del av all information som kommer att ges till forskningspersonen i samband med tillfrågan om deltagande. Både den information som ska ges muntligt och den som ska ges skriftligt. Om vårdnadshavare ska samtycka till deltagande ska även den information som ges till vårdnadshavarna bifogas. Om anhörig ska ges möjlighet att motsätta sig deltagande ska även den information som ges till anhörig bifogas. Informationen ska vara skriven på svenska.*
☐	6	Enkäter, frågeformulär, intervjuguider eller	*Materialet ska vara utformat/skrivet på svenska.*

		intervjufrågor som kommer att användas i projektet.	
☐	7	Variabellista	*Etikprövningsmyndigheten behöver inte alltid en redovisning på variabelnivå för att göra sin bedömning, men det kan ibland underlätta vid begäran hos registerhållaren om den kompletta variabellistan funnits med vid etikprövningen.*
☐	8	Gemensam EU-blankett	*Ska bifogas om forskningen avser klinisk läkemedelsprövning.*
☐	9	Sammanfattning av protokollet på svenska	*Ska bifogas om forskningen avser klinisk läkemedelsprövning.*
☐	10	Prövarhandbok, alternativt bipacksedel, produktresumé eller IB (investigators brochure)	*Ska bifogas om forskningen avser klinisk läkemedelsprövning.*
☐	11	Information om joniserad strålning, stråldosbilaga	*Vid etikprövning av medicinsk, biomedicinsk eller odontologisk forskning som innebär exponering med joniserande strålning ska Etikprövningsmyndigheten fastställa dosrestriktioner för forskningspersoner som inte förväntas få någon direkt medicinsk fördel av exponeringen. Om forskningen innehåller joniserad strålning ska blankett "stråldosbilaga" fyllas i och bifogas ansökan.*
☒	12	CV för ansvarig forskare	*Bifoga CV för ansvarig forskare.*<br>*I undantagsfall kan icke disputerad forskare godtas om annan medverkande disputerad forskare uttalat att forskningen sker under aktivt överinseende av denne. Uttalandet ska vara skriftligt och bifogas. CV för den disputerade ska även bifogas*
☒	13	Övriga bilagor	Studieplan, forskningspersonsinformation och bilaga 1 för EPM-ansökan Dnr. 2020-01479.

UNDERSKRIFT OCH INTYGANDE

1.17. Intygande

I och med att ansökan undertecknas intygar du som är ansvarig forskare samt du som är behörig företrädare följande;

Att den information som lämnas i ansökan om etikprövning och samtliga medföljande bilagor är riktig och fullständig.

Att verksamhetsansvariga i samtliga medverkande verksamheter är informerade om forskningsprojektets innehåll och utförande och att de har samtyckt till att delta i studien.

Att du säkerställt att det i samtliga medverkande verksamheter finns resurser som garanterar forskningspersonernas säkerhet och integritet vid genomförandet av den forskning som beskrivs i ansökan.

Att ansvarig forskare ges rätt att företräda huvudmannen i alla framtida kontakter med Etikprövningsmyndigheten som rör detta forskningsprojekt samt ansöka om ändringar i forskningsprojektet.

Att du tagit del av Etikprövningsmyndighetens information om hantering av personuppgifter på myndighetens webbplats.

Underskrift ansvarig forskare
Joakim Dillner
2020-03-30

Underskrift behörig företrädare för forskningshuvudman
Björn Zoega
2020-03-30

Ansökan om etikprövning
Ansökan om ändring

1.1. **Ange diarienummer på den tidigare godkända grundansökan.**
2020-01620

1.2. **Ange ansvarig forskare för den tidigare godkända grundansökan.**
Joakim Dillner

1.3. **Ange forskningshuvudman för den tidigare godkända grundansökan.**
Region Stockholm

1.4. **Ange titel på den tidigare godkända grundansökan.**

Studier av pågående och genomgången SARS-CoV-2 infektion (som orsakar COVID-19) på akutsjukhus i Stockholms län

1.5. **Beskriv kortfattat den ändring av tidigare godkänd ansökan som planeras.**
Titel ändras till: **Studier av pågående och genomgången SARS-CoV-2 infektion (som orsakar COVID-19) inom vård och omsorg i Stockholms län.**
Tillägg av övriga forskningshuvudmän:
SLSO, Region Stockholm
Södertälje sjukhus, Region Stockholm
Roo Hemtjänst & Vård
Capio S:t Görans sjukhus
S:t Eriks Ögonsjukhus
Tiohundra (inkluderar Norrtälje Sjukhus)
Ersta sjukhus
Tillägg av ytterligare medverkande forskare:
Peter Nilsson, professor, SciLifeLab
Sophia Hober, professor, KTH
Åsa Seigerud, Bitr Verksamhetschef, Roo Hemtjänst & Vård
Clara Hellner, FOU-direktör, SLSO
Carina Lundberg Uudelepp, Regiondirektör, Region Stockholm
Mikael Ohrling, Sjukvårdsdirektör, SLSO
Mikael Runsiö, VD, Södersjukhuset
Yvonne Haglund Åkerlind, VD, Danderyds sjukhus
Peter Holm, VD, Capio S:t Görans sjukhus
Christophe Pedroletti, VD, Södertälje sjukhus
Peter Graf, VD, Tiohundra
Jan-Åke Zetterström, sjukhuschef, Ersta sjukhus
Anders Boman, VD, S:t Eriks Ögonsjukhus
Studiens primära hypotes ändras till: Kan analys av om medarbetare inom vård och omsorg och patienter/brukare exponerats för SARS-CoV-2 möjliggöra en reducerad smittspridning inom vård och omsorg? Kan en trolig effekt av en sådan strategi uppmätas?

1.6. **Ange de skäl som ligger till grund för den planerade ändringen.**
Sedan ansökan skickades in har det framkommit att, förutom det akuta behovet om bättre kunskap om SARS-CoV-2 infektionen inom akutsjukvården, så finns ett minst lika stort behov av kunskap om infektionen inom vård och omsorg utanför akutsjukhusen. Många äldre och sköra som är särskilt känsliga för infektionen vårdas där.

1.7. **Gör en värdering av hur förhållandet mellan riskerna och nyttan av projektet förändras med anledning av den planerade ändringen.**
Vi bedömer att förhållande mellan riskerna och nyttan av projektet inte förändras.

1.8. **Beskriv i förekommande fall hur informationen till forskningspersonerna förändras med anledning av den planerade ändringen.**
Informationen om vilka forskningspersoner som får erbjudande att delta i studien utökas till att även omfatta boende och personal vid närvård, äldreboenden samt hemtjänstpersonal.

1.9. **Beskriv i förekommande fall hur annan information/bilagor förändras med anledning av den planerade ändringen.**

Lista på ändringar i originalansökan bifogas.

1.10. **Ange i förekommande fall vilka bilagor som bifogas ansökan.**

Bilaga 1. Lista på ändringar. Bilaga 2. Reviderad forskningsplan

I och med att ansökan undertecknas intygar du som är ansvarig forskare följande;

Att den information som lämnas i ansökan om etikprövning och samtliga medföljande bilagor är riktig och fullständig.

Att verksamhetsansvariga i samtliga medverkande verksamheter är informerade om forskningsprojektets innehåll och utförande och att de har samtyckt till att delta i studien.

Att du säkerställt att det i samtliga medverkande verksamheter finns resurser som garanterar forskningspersonernas säkerhet och integritet vid genomförandet av den forskning som beskrivs i ansökan.

Att du tagit del av Etikprövningsmyndighetens information om hantering av personuppgifter på myndighetens webbplats.

Underskrift ansvarig forskare (eller behörig företrädare för forskningshuvudman)
Joakim Dillner, professor
2020-04-14

Ansökan om etikprövning – *Bilaga 1*
Beskrivning av forskningsprojektet

2. Typ av forskning

2.1. På vilket eller vilka sätt handlar projektet om forskning enligt 3-4 §§ etikprövningslagen?

- ☒ 3 § 1 Forskningen kommer att samla in känsliga personuppgifter.
- ☐ 3 § 2 Forskningen kommer att samla in personuppgifter om lagöverträdelser.
- ☒ 4 § 1 Forskningen innebär ett fysiskt ingrepp på en forskningsperson.
- ☒ 4 § 2 Forskningen utförs enligt en metod som syftar till att påverka forskningspersonen fysiskt eller psykiskt, eller så innebär forskningen en uppenbar risk att skada forskningspersonen.
- ☒ 4 § 3 Forskningen avser studier på biologiskt material som har tagits från en levande människa och kan härledas tillbaka till denna människa.
- ☐ 4 § 4 Forskningen avser ett fysiskt ingrepp på en avliden människa.
- ☐ 4 § 5 Forskningen avser studier på biologiskt material som tagits från en avliden människa och kan härledas tillbaka till denna människa.

- ☐ Forskningen faller inte under etikprövningslagens tillämpningsområde.

Forskningsperson: De levande människor som forskningen avser.

Personuppgifter: All slags information som direkt eller indirekt kan hänföras till en fysisk person som är i livet.

Känsliga personuppgifter: Känsliga personuppgifter är uppgifter som avslöjar ras eller etniskt ursprung, politiska åsikter, religiös eller filosofisk övertygelse, medlemskap i fackförening, hälsa, en persons sexualliv eller sexuella läggning, genetiska uppgifter och biometriska uppgifter som entydigt identifierar en person.

Personuppgifter om lagöverträdelser: Personuppgifter om lagöverträdelser som innefattar brott, domar i brottmål, straffprocessuella tvångsmedel eller administrativa frihetsberövanden.

2.2. [Om 3 § 1] **Ange vilken typ av känsliga personuppgifter som kommer behandlas i projektet.**

- ☐ ras eller etniskt ursprung
- ☐ politiska åsikter
- ☐ religiös eller filosofisk övertygelse
- ☐ medlemskap i fackförening
- ☒ hälsa
- ☐ en persons sexualliv eller sexuella läggning
- ☐ genetiska uppgifter
- ☐ biometriska uppgifter som entydigt identifierar en person.

Personuppgifter: All slags information som direkt eller indirekt kan hänföras till en fysisk person som är i livet.

Känsliga personuppgifter: Känsliga personuppgifter är uppgifter som avslöjar ras eller etniskt ursprung, politiska åsikter, religiös eller filosofisk övertygelse, medlemskap i fackförening, hälsa, en persons sexualliv eller sexuella läggning, genetiska uppgifter och biometriska uppgifter som entydigt identifierar en person. Enligt definition i EU:s Dataskyddsförordning.

OBS! Om inga känsliga personuppgifter kommer behandlas i projektet så behöver frågan inte besvaras.

3. Syfte och frågeställningar

3.1. Skriv en populärvetenskaplig sammanfattning av forskningsprojektet (max 300 ord).

Beskriv projektet på ett sammanfattande vis. Tänk på att texten ska kunna förstås av personer som inte har vetenskaplig kompetens. Undvik därför terminologi som kräver specialkunskaper.

En global pandemi av SARS-CoV-2 infektion orsakar COVID-19 sjukdom med hög dödlighet över hela världen. Trycket på akutsjukvården är stor och i nuläget har Sverige 214 fall av svårt eller kritiskt sjuka i COVID-19 (källa: www.worldometers.info/coronavirus; 28 mars, 2020, kl. 16.14). Vi har mycket bristande kännedom om hur infektionen sprids i sjukvården och det är helt nödvändig kunskap för att kunna planera en god och patientsäker vård. Vi vill tillfråga personal och patienter vid akutsjukhus i Stockholms län om att delta i en undersökning om de har pågående eller genomgången SARS-CoV-2 infektion, för att förstå hur infektionen sprids i sjukvården.

3.2. Vad är det vetenskapliga syftet med projektet?

Beskriv det övergripande syftet med projektet. Redogör för vad det är för forskningsproblem som projektet ska behandla och vilka avgränsningar som gjorts.

Att mäta och kartlägga förekomsten av SARS-CoV-2 bland sjukhuspersonal och patienter vid akutsjukhus i Stockholms län. Vi har mycket bristande kännedom om hur infektionen sprids i sjukvården och det är helt nödvändig kunskap för att kunna planera en god och patientsäker vård. Hypotesen är att en fullgod planering kommer att bidra till en långsammare smittspridning och minska trycket på sjukvården och därmed göra den mer patientsäker. Studien begränsas till att omfatta Stockholms läns akutsjukhus, där belastningen av COVID-19 patienter är som störst i landet.

3.3. Vilka är de vetenskapliga frågeställningarna?

Ange klart och tydligt den eller de vetenskapliga frågeställningarna i projektet.

Kan analys av om vårdpersonal och patienter exponerats för SARS-Cov-2 möjliggöra en reducerad smittspridningen inom sjukvården? Kan en trolig effekt av en sådan strategi uppmätas?

4. Metod

4.1. Redogör för metod inkl. proceduren, tekniken eller behandlingen.

Det ska framgå hur projektet planeras att genomföras. Beskriv insamlade datas karaktär och ange hur datas tillförlitlighet ska säkerställas.

Om enkäter och intervjuer ingår ska tillvägagångssätt, frågornas innehåll och hur slutsatser dras beskriva. Bifoga enkäter och skattningsskalor.

För medicinsk forskning ska anges t.ex. typer av ingrepp, mätmetoder, antal besök, tidsåtgång, doser, och administrationssätt för eventuella läkemedel.

Om projektet är ett samarbete med utlandet ska det tydligt framgå vilken del av forskningen som utförs i Sverige och vilken del av forskningen som utförs utanför Sverige. Det är endast forskning som ska utföras i Sverige som Etikprövningsmyndigheten kan pröva.

Personal och patienter vid akutsjukhus i Stockholms län tillfrågas om att delta i studien med skriftlig forskningspersonsinformation, varefter skriftligt samtycke inhämtas. För de forskningspersoner som samtycker tas ett prov från näsan (nasofarynx) för påvisning och sekvensbestämning av SARS-CoV-2, med kvantitativ PCR samt sekvenering vid Karolinska Universitetslaboratoriet. Genomgången SARS-CoV-2 infektion undersöks genom att mäta förekomsten av SARS-CoV-2 antikroppar i serum med hjälp av de metoder som idag finns tillgängliga, Luminex (Immunoassay), Immunofluoresens och ELISA. För detta tas ett blodprov om 5 milliliter. Serumanalyser utförs företrädesvis vid Karolinska Universitetslaboratoriet. Provtagning utförs av utbildad personal i tillfälliga provtagningslokaler uppsatta utanför respektive sjukhus. En samlad databas innehållande namn, personnummer, vårdavdelning, provtagningsdatum samt resultat av alla Corona-analyser upprättas vid Karolinska Universitetslaboratoriet, där den förvaras under skalskydd och vårdsekretess. Forskningspersonerna delges provsvar och dess tolkning via provsvarsmodulen inom 1177 Vårdguiden (1177 PEP - patientens egen provtagning). Vid positivt svar (förekomst av SARS-CoV-2) delges uppgiften ansvarig Smittskyddsläkare för handläggning enligt vid tidpunkten gällande rekommendationer (dessa kan ändras över tid men har fram till nu varit isolering i 14 dagar eller vid allvarligare symptom uppsöka vård).

4.2. Redogör för på vilket sätt metoden skiljer sig från klinisk rutin eller den ordinarie behandlingen.

Ange vad som avviker eller tillkommer med anledning av forskningsprojektet.

Provtagningen sker enligt klinisk rutin. Proven analyseras med ej ackrediterade metoder då det i dagsläget inte finns ackrediterade metoder för analys av SARS-CoV-2 eller immunitet mot SARS-CoV-2. För att öka tillförlitligheten till provresultaten utförs tre olika analyser parallellt och provresultaten sammanvägs.

4.3. Redogör för tidigare erfarenheter (egna och/eller andras) av den använda proceduren, tekniken eller behandlingen.

Redogör för vilken erfarenhet och kompetens som medverkande forskare har av att använda den procedur, teknik eller behandling om man planerar att använda i projektet. Redogör även för vilken erfarenhet som finns generellt eller globalt.

Samtliga provanalyser planeras genomföras vid eller på uppdrag av Karolinska Universitetslaboratoriet (KUL). KUL utför närmare 20 miljoner labanalyser per år.

5. Tidsplan

5.1. Förväntat startdatum för projektet:

Ange om möjligt, beräknad start av projektet.

2020-04-06 eller det datum då tillstånd ges.

5.2. Förväntat slutdatum för projektet:

Ange om möjligt, beräknat slut av projektet.

2020-12-31

5.3. Tidsplan för de olika delar som ingår i projektet:

Uppbyggnad av provtagningsstationer och själva provtagningen förväntas ske under de två första veckorna av projektet. Parallellt med och en vecka efter provtagningen sker provanalyseringen och utsvar till forskningspersonerna. Den kliniska delen av studien förväntas vara klar inom den närmasta månaden, men analys av studieresultat kan ta längre tid.

Redogör för projektets tidsschema, hur de olika delarna förhåller sig till varandra, vid behov kan ett förklarande flödesschema bifogas.

6. Datainsamling

6.1. Redogör för datainsamling och datas karaktär.

Redogör för hur datainsamlingen ska gå till. Beskriv den data som ska samlas in och hur den ska samlas in.

All data samlas inom befintliga dokumentationssystem i vården.

6.2. Redogör för det statistiska underlaget för studiepopulationen/ undersökningsmaterialets storlek.

Redogör för de beräkningar och överväganden som gjorts för att komma fram till hur många deltagare som behövs för att uppnå tillräcklig statistisk styrka. Redogörelsen av hur man kommit fram till lämpligt urval ska beskriva projektets möjligheter att besvara frågeställningarna.

Då vi i dagsläget inte vet hur stor smittspridningen bland sjukhuspersonal och patienter är kan inga beräkningar av den statistiska styrkan göras. Studiepopulationens storlek har bestämts baserat på praktiska och etiska överväganden av genomförbarheten av studien.

6.3. Hur kommer undersökningsprocedurerna att dokumenteras?

Redogör för hur undersökningarna och eventuella ingrepp dokumenteras. Ange om band- och videoinspelningar kommer användas.

Undersökningarna och behandlingarna dokumenteras med sedvanlig vårddokumentation.

6.4. Hur kommer insamlad data att hanteras och förvaras?

Redogör för hur data kommer att hanteras efter insamlingen. Om data kommer att pseudonymiseras (”kodas”) ska kodningsförfarandet beskrivas. Det ska framgå var kodlistorna/kodnycklarna kommer att förvaras samt vem/vilka som kommer ha tillgång till dem. Det ska även framgå hur länge data kommer att sparas samt om det kommer att avidentifieras (genom att kodnyckel förstörs) eller förstöras i sin helhet.

All datahantering och dokumentation sker inom sjukvårdens datasystem och under vårdsekretess.

7. Etiska överväganden

7.1. Vilka risker kan ett deltagande medföra för de forskningspersoner som ingår i forskningsprojektet?

Risker kan vara av olika karaktär. Det kan exempelvis vara fysisk eller psykisk skada, smärta, obehag, integritetsintrång på kort eller lång sikt. Ange vilka risker det kan finnas för de forskningspersoner som deltar i detta forskningsprojekt.

Forskningspersonerna kan tänkas känna det svårt att tacka nej på grund av grupptryck när kollegor tackar ja. Det kan också tänkas finnas integritetsrisker då provtagning sker i en relativt öppen miljö. All information och provtagning sker dock under vårdsekretess. Provtagningen kan upplevas som obehaglig och smärtsam och blodutgjutning kan förekomma vid insticksstället för blodprovtagningen.

7.2. Vilken nytta kan ett deltagande medföra för de forskningspersoner som ingår i forskningsprojektet?

Ange vilken hjälp de forskningspersoner som deltar i detta forskningsprojekt kan få som ett resultat av forskningsprojektet.

Nyttan på kort sikt är att forskningspersonerna får svar på om de bär på SARS-CoV-2 eller inte eller om de genomgått en coronainfektion. Det får till följd att forskningspersoner som är coronavirus-bärare isoleras vilket är till gagn för samtliga forskningpersoner och hela populationen. På lång sikt gynnas hela samhället av att planering av en god och säker vård förbättras och av att smittspridningshastigheten kan mattas av.

7.3. Gör en värdering av förhållandet mellan riskerna och nyttan av projektet.

Forskning får enligt etikprövningslagen bara godkännas om riskerna som den kan medföra för forskningspersonernas hälsa, säkerhet och personliga integritet uppvägs av dess vetenskapliga värde.

Deltagandet i forskningsstudien innebär att provtagning görs vid ett tillfälle enligt välbeprövade kliniska metoder vars biverkningar är av lindrig karaktär. Risken för integritetsintrång vid provtagningstillfället och efterföljande provanalys anses ringa då analysdata hanteras inom sjukhusens slutna datasystem med skalskydd och vårdsekretess av utbildad personal. Projektet förväntas generera en kunskapsbas som kan användas för att förstå hur infektionen sprids i sjukvården och för att kunna planera en god och säker vård. Nyttan bedöms därför överväga de risker som forskningspersonerna i studien utsätts för.

7.4. Beskriv hur projektet har utformats för att minimera riskerna för forskningspersonerna.

Forskning får enligt etikprövningslagen bara godkännas om det förväntade resultatet inte kan nås på ett annat sätt som innebär mindre risker för forskningspersoners hälsa, säkerhet och personliga integritet.

Behandling av personuppgifter som avses i 3 § får godkännas bara om den är nödvändig för att forskningen ska kunna utföras.

Observera att om en behandling ska prövas för första gången på människa måste detta tydligt framgå och relevanta säkerhetsrutiner tydligt beskrivas.

Ange vilka risker som ett deltagande i forskningsprojektet kan innebära. Finns det risker t.ex. med att ingå i en kontrollgrupp?

För att minimera riskerna för forskningspersonerna samlas all forskningsdata i en databas under skalskydd och vårdsekretess.

7.5. Identifiera och precisera om eventuella etiska problem (nackdelar/fördelar) kan uppstå i ett vidare perspektiv genom forskningsprojektet.

Redovisa t.ex. om vissa grupper (andra än de forskningspersoner som ingår i forskningsprojektet) kan komma att utpekas respektive få hjälp som ett resultat av projektet. Frågan avser även de indirekta riskerna eller den indirekta nyttan. T.ex. genetisk påverkan på kommande generationer eller om resultaten på annat sätt kan tänkas skada vissa grupper.

Om analyserna visar sig ha förväntad effekt på bättre kontroll på smittspridning och bättre möjlighet att ge en god och säker vård så kan efterfrågan/krav på testning för SARS-CoV-2 öka kraftigt och det är inte säkert att det finns möjlighet att tillgodose detta. Kan leda till etisk problematik om vilka analyser som bör prioriteras.

8. Forskningspersoner

8.1. Hur görs urvalet av forskningspersoner?

Forskningsperson: De levande människor som forskningen avser.

Beskriv vilka forskningspersoner som kommer inkluderas i projektet. Redogör för de överväganden som gjorts vid valet av forskningspersoner. Om vissa grupper utesluts från deltagande i projektet ska en motivering till uteslutandet framgå.

Redogör även för hur forskaren kommer i kontakt med eller får kännedom om lämpliga forskningspersoner.

Personal och patienter vid Stockholms läns akutsjukhus som inte redan testats positivt för SARS-CoV-2 eller nyligen provtagits kommer att tillfrågas att delta i studien av en av sjukhuset utsedd kontaktperson. Endast myndiga personer upp till en ålder av 99 år kommer att tillfrågas.

8.2. Hur många forskningspersoner kommer att inkluderas i forskningsprojektet?

Ange hur många forskningspersoner som totalt kommer inkluderas i projektet samt i förekommande fall hur många som kommer inkluderas i olika delprojekt.

Personal och patienter vid akutsjukhusen i Stockholms län kommer att tillfrågas om att delta. Vi uppskattar det till cirka 20000 personer.

8.3. Vilka urvalskriterier kommer att användas för inklusion?

Ange vilka kriterier som måste uppfyllas för att en forskningsperson ska inkluderas i projektet.

Urvalskriterier för inklusion är (alla tre uppfyllda):

- Inlagd eller anställd vid ett akutsjukhus i Stockholms län
- Mellan 18-99 år
- Lämnat samtycke att delta i studien

8.4. Vilka urvalskriterier kommer att användas för exklusion?

Ange vilka kriterier som måste uppfyllas för att en forskningsperson ska exkluderas ur projektet.

Urvalskriterier för exklusion är

- de som inte lämnat informerat samtycke till att delta i studien
- de med redan bekräftad SARS-CoV-2 infektion eller som nyligen provtagits för SARS-CoV-2 infektion

8.5. Ange relationen mellan forskare och forskningspersonerna.

Ange forskarens roll i relation till forskningspersonens roll. Det kan t.ex. vara som behandlare (läkare, psykolog, fysioterapeut etc.) och patient/klient, som lärare och student eller arbetsgivare och anställd. All form av relation som kan tänkas medföra risk för påverkan ska beskrivas. Enligt etikprövningslagen ska information och samtycke ägnas särskild uppmärksamhet om forskningspersonen står i ett beroendeförhållande till huvudmannen, forskaren eller antas ha svårigheter att ta tillvara sin rätt.

Då forskningsprojektet genomförs vid Stockholms läns akutsjukhus kommer även forskarna själva samt deras kollegor och patienter utgöra forskningspersoner. På grund av studiens omfattning anses risken för påverkan att delta eller inte delta i studien liten.

8.6. Vilket försäkringsskydd finns för de forskningspersoner som deltar i forskningsprojektet?

Forskningshuvudmannen har ansvar för att kontrollera att det finns försäkring som täcker eventuella skador som kan uppkomma i samband med forskningen. Ange vilka försäkringar som forskningspersonen kommer att omfattas av samt vilket skydd försäkringarna ger forskningspersonen.

Projektet genomförs inom Region Stockholms verksamhet, där sedvanlig patientförsäkring gäller.

8.7. Redogör för den beredskap som finns för att hantera oväntade bifynd eller händelser under forskningsprocessen som kan äventyra forskningspersonernas säkerhet.

Beskriv vilken tillgång forskningsprojektet har till utrustning, personal och kompetens för att hantera eventuellt oväntade komplikationer eller bifynd. Beskriv även vilken planering som finns för att hantera eventuella oväntade komplikationer eller bifynd.

Personer med höga nivåer av antikroppar kommer att tillfrågas om de vill donera blod för en separat studie av om plasma från tillfrisknade personer har effekt som behandling vid svår COVID-19 sjukdom (Studieplan, forskningspersonsinformation och bilaga 1 för den EPM-ansökan medsändes som bilaga till denna ansökan).

8.8. Kommer ekonomisk ersättning eller andra förmåner betalas ut till forskningspersonerna?

Forskningspersonerna kan utöver ersättning för resor, förlorad arbetsinkomst eller andra utgifter erhålla viss ersättning för obehag och besvär. Ersättningen ska vara skälig. Om barn eller ungdomar under 18 år deltar i forskningsprojektet får sådan ersättning inte vara stor och den bör inte erbjudas i samband med rekryteringen. Vid klinisk läkemedelsprövning med barn eller ungdomar under 18 år får inga incitament eller ekonomiska förmåner ges, undantaget kostnadsersättningar.

Nej

8.8.1. [Om Ja 8.8] Vilken ekonomisk ersättning kommer betalas ut och när?

9. Information och samtycke

9.1. Kommer forskningspersonerna att informeras om forskningsprojektet och tillfrågas om de vill vara med eller inte?

Grundregeln, enligt etikprövningslagen, är att forskning bara utförs om forskningspersonen har informerats och samtyckt till deltagande. Information till forskningspersonerna kan ges både muntligt och skriftligt.

Ja

9.1.1. [Om Ja 9.1] Hur, när (i vilket skede) och av vem informeras och tillfrågas forskningspersonerna?

Beskriv proceduren för hur information ges och samtycke inhämtas. Vem som frågar, när detta sker och hur samtycket dokumenteras. På vilket sätt säkerställs att forskningspersonen ges betänketid och möjlighet att ställa frågor.

Utförlig redovisning är särskilt viktig när det ingår barn eller personer med nedsatt beslutskompetens i forskningsprojektet.

Forskningspersonerna informeras om studien i samband med att de blir kontaktade och tillfrågade om de vill delta i forskningsstudien. Vid provtagningstillfället får de ta del av skriftlig forskningspersonsinformation och lämna informerat samtycke innan provtagning.

9.1.2. [Om Nej 9.1] Motivera varför forskningspersonerna inte ska informeras och tillfrågas.

Lämna en utförlig redogörelse för de avvägningar som har gjorts och de skäl som ligger till grund för bedömningen att forskningspersonerna inte ska informeras och tillfrågas. Observera att forskning utan information och samtycke endast är möjlig i undantag fall och i den forskning sam avses i 3 § etikprövningslagen, eller 20–22 § i etikprövningslagen.

Max 4500 tecken

9.2. Kommer barn under 18 år att ingå i forskningsprojektet?

För barn under 15 år måste barnets samtliga vårdnadshavare ge sin tillåtelse (samtycka) till att barnet får delta i forskningsprojektet.

Barn mellan 15 och 18 år, som inser vad forskningen innebär för hans eller hennes del, ska informeras om och lämna eget samtycke till forskningen. I andra fall ska barnets samtliga vårdnadshavare ge sin tillåtelse (samtycka) till att barnet får delta i forskningsprojektet.

Forskningen får, trots vårdnadshavarnas tillåtelse (samtycke) inte utföras om barnet motsätter sig att forskningen utförs.

Nej

9.2.1. [Om Ja 9.2] Ange barnens ålder.

9.3. Kommer forskningspersoner, vars mening på grund av sjukdom, psykisk störning, försvagat hälsotillstånd eller något annat liknande förhållande inte kan inhämtas, att ingå i forskningsprojektet?

Ange om forskningsprojektet kommer involvera personer som själva inte kan samtycka till sitt eget deltagande p.g.a. sjukdom, psykisk störning, försvagat hälsotillstånd eller liknande tillstånd.

Nej

9.3.1. [Om Ja 9.3] **Motivera varför denna grupp av forskningspersoner ska ingå i projektet.**

Forskning utan samtycke på denna grupp av forskningspersoner får endast utföras om forskningen förväntas ge en kunskap som inte är möjlig att få genom forskning med samtycke.

Forskningen ska dessutom förväntas leda till direkt nytta för forskningspersonen. Alternativt ska forskningen bidra till ett resultat som kan vara till nytta för forskningspersonen eller annan som lider av samma eller liknande sjukdom eller tillstånd samt innebära en obetydlig risk för skada eller obehag för forskningspersonen. Forskningspersonen ska så långt som möjligt informeras personligen om forskningen.

Samråd ska ske med närmaste anhörig. Samråd ska också ske med god man eller förvaltare om frågan ingår i uppdraget som god man eller förvaltare. Forskningen får inte utföras om forskningspersonen i någon form ger uttryck för att inte vilja delta eller om någon av dem som samråd har skett med motsätter sig införandet.

9.3.2. [Om Ja 9.3] **Beskriv hur samråd med närmaste anhörig, god man eller förvaltare kommer att ske.**

Vid forskning på forskningspersoner, vars mening inte går att inhämta, ska samråd ske med närmaste anhörig som ska få möjlighet att motsätta sig deltagandet. Samråd ska ske med god man eller förvaltare om frågan ingår i uppdraget som god man eller förvaltare.

10. Registeruppgifter

10.1. Kommer projektet att begära ut uppgifter från ett befintligt register?

Här avses alla typer av register som innehåller personuppgifter eller uppgifter som tidigare varit personuppgifter men senare avidentifierats.

Nej

10.1.1. [Om Ja 9.1] **Ur vilket eller vilka register kommer uppgifterna att begäras?**

Namnge registret eller registren som uppgifterna kommer att begäras ifrån. Ange även huvudman för respektive register.

Sjukhusens administrativa databaser kommer att användas, men då dataanalys görs i sjukhusens regi är det inte fråga om utlämning.

10.1.2. [Om Ja 9.1] **Vilka uppgifter kommer att begäras ut och varför?**

Beskriv vilken typ av uppgifter som kommer att begäras ut och varför de behövs för att besvara projektets frågeställningar.

En komplett variabellista kan med fördel bifogas som bilaga. Etikprövningsmyndigheten behöver inte alltid en redovisning på variabelnivå för att göra sin bedömning, men det kan ibland underlätta vid begäran hos registerhållaren om den kompletta variabellistan funnits med vid etikprövningen.

När man varit i tjänst och var, bostadsadress. Inläggningsdatum, sjukdomsdiagnos och medicinska åtgärder som vidtagits.

11. Resultat från djurförsök

11.1. Finns det relevanta resultat från djurförsök?

Frågan avser framförallt klinisk behandlingsforskning. Om djurförsök inte har utförts, ange då anledningen till detta.

Ej Aktuellt

11.1.1. [Om Ja 11.1] Redogör för resultaten av djurförsöken

Redogör övergripande för de djurförsök som utförts och vilka resultat de gav. Framförallt ska de resultat som är av relevans för detta forskningsprojekt redogöras för.

12. REDOVISNING AV RESULTAT

12.1. Hur garanteras tillgång till data för forskningshuvudmannen och medverkande forskare?

Normalt ska den som ansvarar för genomförandet av forskningen ha full tillgång till data. Om flera forskare samverkar i uppdragsforskning bör den forskare som är huvudansvarig för genomförandet i förväg komma överens med övriga forskare om tillgång till data.

Alla data hanteras av forskningshuvudmannen och medverkande forskare, som alla har anställning hos fotskningshuvudmannen och arbetar under vårdsekretess.

12.2. Vem eller vilka ansvarar för databearbetning och skriftlig redovisning av resultaten?

Ange vem eller vilka som kommer bearbeta och analysera forskningsdata och vem eller vilka som kommer utforma den skriftliga redovisningen. Normalt ska den som ansvarar för genomförandet av forskningen ha full tillgång till data.

Huvudansvarig forskare tillsammans med medverkande forskare ansvarar för databearbetning och skriftlig redovisning av resultaten.

12.3. Hur och när planeras resultaten att offentliggöras?

Ange i vilken form resultaten planeras att offentliggöras. Exempelvis vetenskaplig publicering med peer review, open acess, intern rapport. Ange om möjligt en tidsplan för offentliggörandet.

Huvudresultatet kommer att publiceras direkt på internet samt uppföljas med publicering i vetenskapliga artiklar och presenteras på konferenser samt kommuniceras till beslutsfattare i frågan

12.4. På vilket sätt garanteras forskningspersonernas rätt till integritet när materialet offentliggörs?

Redogör för hur data presenteras när den offentliggörs och hur forskningspersonernas integritet skyddas vid offentliggörandet.

Data redovisas enbart i aggregerad form, utan att kunna hänföras till enskild individ.

13. EKONOMISKA FÖRHÅLLANDEN

13.1. Redovisa eventuella ekonomiska överenskommelser med bidragsgivare eller andra finansiärer (namn och belopp).

Redovisa alla överenskommelser om finansiering som har slutits med den eller de som ska genomföra forskningen. Ange vilka belopp som kommer att erhållas för forskningsprojektet och vad ersättningen ska täcka. Ange även eventuella belopp per forskningsperson.

Studien genomförs inom ordinarie hälso och sjukvård.

13.2. Redovisa forskningshuvudmannens, huvudansvarig forskares och medverkande forskares egna ekonomiska intressen.

Redovisa egna ekonomiska intressen i form av t.ex. aktieinnehav, anställning, konsultuppdrag i finansierade företag, eget företag som kan få direkt eller indirekt vinst av forskningen.

Samtliga medverkande forskare har inga dylika intressen att redovisa för denna studie.

Bilaga 1

Lista på ändringar till etiktillstånd dnr. 2020-01620

I ansökan

Punkt 1.1: titel på forskningsprojektet ändras till ”**Studier av pågående och genomgången SARS-CoV-2 infektion (som orsakar COVID-19) inom vård och omsorg i Stockholms län”.**

Punkt 1.2: antalet forskningshuvudmän ändras till flera.

Punkt 1.8: övriga forskningshuvudmän ändras till:

SLSO, Region Stockholm

Södertälje sjukhus, Region Stockholm

Roo Hemtjänst & Vård

Capio S:t Görans sjukhus

S:t Eriks Ögonsjukhus

Tiohundra (inkluderar Norrtälje Sjukhus)

Ersta sjukhus

Punkt 1.11: ytterligare medverkande forskare:

Peter Nilsson, professor, SciLifeLab

Sophia Hober, professor, KTH

Åsa Seigerud, Bitr Verksamhetschef, Roo Hemtjänst & Vård

Clara Hellner, FOU-direktör, SLSO

Carina Lundberg Uudelepp, Regiondirektör, Region Stockholm

Mikael Ohrling, Sjukvårdsdirektör, SLSO

Mikael Runsiö, VD, Södersjukhuset

Yvonne Haglund Åkerlind, VD, Danderyds sjukhus

Peter Holm, VD, Capio S:t Görans sjukhus

Christophe Pedroletti, VD, Södertälje sjukhus

Peter Graf, VD, Tiohundra

Jan-Åke Zetterström, sjukhuschef, Ersta sjukhus

Anders Boman, VD, S:t Eriks Ögonsjukhus

Bilaga 1

Punkt 3.1, 3.2, 4.1, 8.1, 8.2, 8.3: ytterligare medverkande organisationer och genom dem rekryterad medarbetare, patienter och brukar inom vård och omsorg i Stockholms län.

Bilaga 1

Punkt 3.3: de vetenskapliga frågeställningarna ändras till: Kan analys av om medarbetare inom vård och omsorg och patienter/brukare exponerats för SARS-CoV-2 möjliggöra en reducerad smittspridning inom vård och omsorg? Kan en trolig effekt av en sådan strategi uppmätas?

Punkt 8.2: antalet forskningspersoner som kommer inkluderas i projektet ändras till: 40,000 personer

Bilaga 3

Under syfte, studieupplägg och metodologi, studiepopulation, kriterier och studieobjekt utökas deltagande forskningspersoner att inkludera medarbetare, patienter och brukare inom vård och omsorg i Stockholms län.

Studiebeskrivning: Ändras från akutsjukhus till vård och omsorg.

Datainsamling: ”Motsvarande databaser finns på alla akutsjukhusen” ändras till ”motsvarande databaser finns hos länets arbetsgivare inom vård och omsorg”.

Bilaga 5

Deltagande forskningspersoner och information vem som får erbjudande att delta i studien utökas till personal och patienter/brukare inom vård och omsorg i Stockholms län.

Ansökan om etikprövning – *Bilaga 2*
Beskrivning av biologiskt material

14. Beskrivning av biologiskt material

14.1. Kommer biologiskt material från människor att nyinsamlas för projektet?

Frågan avser ny insamling av biologiskt material (prover) från människor, dvs. material som samlas in specifikt för detta forskningsprojekt.

Ja

14.1.1. [Om ja 14.1] Vilken eller vilka typer av biologiskt material ska samlas in?

Ange vilken eller vilka typer av biologiskt material som ska samlas in för detta forskningsprojekt. Om alternativet Annat biologiskt material väljs ska dessa anges.

☐ Vävnad
☒ Blod
☒ Annat biologiskt material, ange vad

Nasofarynxprov

14.1.2. [Om ja 14.1] Kommer det biologiska materialets ursprung gå att härleda till en enskild person, det vill säga går det att spåra eller koppla samman det biologiska materialet med den individ som materialet kommer ifrån?

Det går att koppla samman det biologiska materialet med individen på två sätt: Antingen direkt (proverna har då märkts med identifierande uppgifter som initialer, födelsenummer eller liknande) eller genom en kodnyckel (materialet är då pseudonymiserat). Om det inte går att koppla samman det biologiska materialet med individen är materialet avidentifierat.

Ja

14.1.2.1. [Om ja 14.1.2] Ange hur det biologiska materialet kommer att kodas.

När ett material kodas ersätts individens identitet på materialet med en kod, och en kodnyckel tas fram. Genom kodnyckeln går det att identifiera vilken individ materialet kommer ifrån.

Proverna ges ID-nummer. ID-numret läggs in i databas som upprättas vid Karolinska Universitetslaboratoriet, där behörig personal har möjlighet att knyta det till forskningspersonens identitet.

14.1.3. [Om ja 14.1] Hur mycket biologiskt material är planerat att samlas in?

Ange antalet prover, antalet bitar och/eller volym som ska samlas in vid varje enskilt provtagningstillfälle. Ange även det totala antalet prover, antalet bitar och/eller den totala volymen för studien. Ange i förekommande fall även antal prover, bitar och/eller volym för varje delstudie.

Ett nasofarynxprov och ett blodprov om 5 milliliter tas vid ett tillfälle.

14.1.4. [Om ja 14.1] Hur länge ska det biologiska materialet vara tillgängligt för projektet?

Ange under vilken tid forskningsprojektet kommer ha tillgång till det biologiska materialet.

2020-04-06 – 20300405

14.1.5. [Om ja 14.1] Kommer det biologiska materialet analyseras och förstöras inom sex månader efter provtagningstillfället, det vill säga är undantagsregeln tillämplig?

Den 1 januari 2019 infördes en ny undantagsregel gällande prov som tas för forskning men som inte ska sparas i biobank. Undantagsregeln innebär att biobankslagen inte är tillämplig på prov som är avsedda för forskning och som analyseras inom sex månader efter provtagningstillfället och förstörs omedelbart efter analysen. Båda villkoren måste uppfyllas.

Även om undantagsregeln blir tillämplig i aktuellt forskningsprojekt ska alla frågor om biologiskt material besvaras med undantag för frågorna om namn och huvudman för biobanken.

Nej

14.1.6. [Om ja 14.1] **Ange en motivering till valet av tid som projektet kommer ha tillgång till det biologiska materialet.**

Erfarenheter av tidigare epidemier är att de ofta kommer i flera vågor, ibland under flera år. Det kan därför vara viktigt att kunna ta fram tidigare prov för parallell analys med nytagna prover. Analysmetoderna förändras snabbt och redan om något år är det troligen så stora skillnader att resultat från tidigare analyser inte kan jämföras med resultat från nya analyser på ett tillräckligt pålitligt sätt. Slutligen avser vi att publicera studien och källmaterial från publicerade studier bör vara bevarade i 10 års tid som en säkerhet för att förebygga forskningsfusk.

14.1.7. [Om ja 14.1] **Hur länge ska det biologiska materialet vara tillgängligt efter att projektet är avslutat?**

Ange under vilken tid sökanden planerar att ha tillgång till det biologiska materialet.

20200406 – 20300405

14.1.8. [Om ja 14.1] **Ange en motivering till valet av tid som projektet kommer ha tillgång till det biologiska materialet efter att projektet avslutats.**

Materialet sparas för eventuell omanalys, dock längst till och med 2030-04-05

14.1.9. [Om ja 14.1] **Ange namnet på den biobank som ska ansvara för provsamlingen.**

Här avses den biobank som kommer att ansvara för den provsamling som bildas under studien, dvs. den samling som det biologiska materialet hämtas till. Ange om möjligt IVO:s registreringsnummer.

Biobank: *En samling biologiskt material från människor, förvarat för ett eller flera ändamål, samt information om detta material. En biobank kan innehålla flera provsamlingar.*

Stockholms Medicinska Biobank

14.1.10. [Om ja 14.1] **Ange huvudman för biobanken**

Ange huvudman för den biobank som ska ansvara för provsamlingen.

Huvudman: den myndighet eller organisation som juridiskt och ekonomiskt har ansvaret för viss verksamhet.

Region Stockholm

14.1.11. [Om ja 14.1] **Vilka analyser kommer att utföras på det biologiska materialet?**

Redogör för alla analyser och bearbetningar som det biologiska materialet ska genomgå.

Kvanitativ PCR, cDNA-sekvenering, Luminex (immunoassay), Immunofluoresens och ELISA.

14.1.12. [Om ja 14.1] **Var kommer analyserna att utföras?**

Ange var analyserna på det biologiska materialet kommer att ske. Om analyser ska ske utanför Sverige ska land/länder anges.

Olika regler gäller om prover skickas för analys inom Sverige, inom EU/ESS eller utanför EU/ESS till så kallat tredje land.

☒ I den egna verksamheten
☒ Inom Sverige
☐ Utanför Sverige, ange land/länder

Vi avser i första hand att utföra alla analyser i egen regi. För serologiska analyser sker en snabb utveckling och vi kan behöva uppdra åt samarbetande laboratorier i Sverige att utföra analyserna

14.1.13. [Om ja 14.1] **Hur kommer det biologiska material att hanteras när analyserna är genomförda?**

Prover i en biobank som har lämnats för analys inom eller utom landet ska lämnas tillbaka till biobank i Sverige eller förstöras (kasseras) när de inte längre behövs för ändamålet. I normalfallet bör det ske inom några få år.

☐ Materialet kommer att förstöras.
☒ Materialet kommer att skickas tillbaka till biobank i Sverige.

14.2. Planerar projektet att använda biologiskt material från människor från en eller flera befintliga provsamlingar?

Frågan avser om man för detta forskningsprojekt kommer att begära tillgång till biologiskt material eller prover som har samlats in tidigare.

Nej

14.2.1. [Om ja 14.2] Vilket biologiskt material är planerat att användas?

Ange vilken typ av biologiskt material som ska användas i detta forskningsprojekt. Om alternativen "annat biologiskt material" väljs ska dessa anges.

- ☐ Vävnad
- ☐ Blod
- ☐ Annat biologiskt material, ange vad

[Om annat] – Max 100 tecken

14.2.2. [Om ja 14.2] Hur mycket biologiskt material är planerat att användas?

Ange antalet prover, bitar och/eller volymen för allt biologiskt material som är planerat att hämtas in och användas för detta projekt.

Max 2000 tecken

14.2.3. [Om ja 14.2] Ange namnet/namnen på ansvariga biobanker och huvudmän som det biologiska materialet kommer att hämtas ifrån.

Samtliga biobanker och huvudmän som det befintliga biologiska materialet kommer att hämtas eller begäras ut ifrån ska namnges.

Biobank: En samling biologiskt material från människor, förvarat för ett eller flera ändamål samt information om detta material. En biobank kan innehålla flera provsamlingar.

Max 2000 tecken

14.2.4. [Om ja 14.2] Ange namnet på den biobank som kommer att ansvara för denna provsamling.

Här avses den biobank som kommer att ansvara för den nya provsamling som bildas, dvs. den samling som det biologiska materialet hämtas till. Ange om möjligt IVO:s registreringsnummer.

Biobank: En samling biologiskt material på människor, förvarat för ett eller flera ändamål samt information om detta material. En biobank kan innehålla flera provsamlingar.

Max 200 tecken

14.2.5. [Om ja 14.2] Ange huvudman för biobanken.

Ange huvudman för den biobank som ska ansvara för den nya provsamlingen som bildas.

Huvudman: Den myndighet eller organisation som juridiskt och ekonomiskt har ansvaret för viss verksamhet.

Max 200 tecken

14.2.6. [Om ja 14.2] Ange hur materialet är kodat.

När ett material kodas ersätts individens identitet på materialet med en kod, och en kodnyckel tas fram. Genom kodnyckeln går det att identifiera vilken individ materialet kommer ifrån.

Max 2000 tecken

14.2.7. [Om ja 14.2] Vilka analyser kommer att utföras på det biologiska materialet?

Redogör för alla analyser och bearbetningar som det biologiska materialet ska genomgå.

Max 2000 tecken

14.2.8. [Om ja 14.2] Var kommer analyserna att utföras?

Ange var analyserna på det biologiska materialet kommer att ske. Om analyser ska ske utanför Sverige ska land/länder anges.

Olika regler gäller om prover skickas för analys inom Sverige, inom EU/ESS eller utanför EU/ESS till så kallat tredje land.

- ☐ I den egna verksamheten
- ☐ Inom Sverige
- ☐ Utanför Sverige, ange land/länder

[Ange land/länder] – Max 500 tecken

14.2.9. [Om ja 14.2] **Hur kommer det biologiska materialet att hanteras när analyserna är genomförda?**

Prover i en biobank som har lämnats för analys inom eller utom landet ska lämnas tillbaka till biobank i Sverige eller förstöras (kasseras) när de inte längre behövs för ändamålet. I normalfallet bör det ske inom några få år.

☐ Materialet kommer att förstöras.
☐ Materialet kommer att skickas tillbaka till biobank i Sverige.

14.2.10. [Om ja 14.2] **Hur länge ska det biologiska materialet vara tillgängligt för projektet?**

Ange under vilken tid det biologiska materialet kommer att sparas för det här forskningsprojektet.

Från och med dag månad år – Till och med dag månad år

14.2.11. [Om ja 14.2] **Ange en motivering till valet av tid som det biologiska materialet kommer vara tillgängligt för projektet.**

Max 2000 tecken

14.2.12. [Om ja 14.2] **Hur länge ska det biologiska materialet vara tillgänglig efter att projektet är avslutat?**

Ange under vilken tid sökanden planerar att ha tillgång till det biologiska materialet.

Från och med dag månad år – Till och med dag månad år

14.2.13. [Om ja 14.2] **Ange en motivering till valet av tid som det biologiska materialet ska vara tillgänglig efter att projektet är avslutat.**

Max 2000 tecken

Forskningsplan

Studier av pågående och genomgången SARS-CoV-2 infektion (som orsakar COVID-19) inom vård och omsorg i Stockholms län

Etikprövningsbeslut

EPM 2020-01620 samt pågående ändring ansökt om

Medverkande forskare

Kalle Conneryd Lundgren, Operativ direktör, Karolinska Universitetssjukhuset

Joakim Dillner, FoU-Chef, Professor, Karolinska Universitetslaboratoriet, Karolinska Universitetssjukhuset

Lars Engstrand, Professor, Karolinska Universitetslaboratoriet, Karolinska Universitetssjukhuset

Lars I Eriksson, FoU-Chef, Professor, ME Intensivvård, Karolinska Universitetssjukhuset

Peter Nilsson, professor, SciLifeLab

Sophia Hober, professor, KTH

Åsa Seigerud, Bitr Verksamhetschef, Roo Hemtjänst & Vård

Clara Hellner, FOU-direktör, SLSO

Carina Lundberg Uudelepp, Regiondirektör, Region Stockholm

Mikael Ohrling, Sjukvårdsdirektör, SLSO

Mikael Runsiö, VD, Södersjukhuset

Yvonne Haglund Åkerlind, VD, Danderyds sjukhus

Peter Holm, VD, Capio S:t Görans sjukhus

Christophe Pedroletti, VD, Södertälje sjukhus

Peter Graf, VD, Tiohundra

Jan-Åke Zetterström, sjukhuschef, Ersta sjukhus

Anders Boman, VD, S:t Eriks Ögonsjukhus

Studiefakta

Typ av studie	Observationsstudie
Studiedesign	Kohortundersökning<br>Öppen studie
Inkludering ålder	18-99
Inkludering kön	Män och Kvinnor

Beskrivning

Bakgrund

Forskningsplan

En global pandemi av SARS-CoV-2 infektion orsakar COVID-19 sjukdom med hög dödlighet över hela världen. Vi har mycket bristande kännedom om hur infektionen sprids inom vård och omsorg och det är helt nödvändig kunskap för att kunna planera en god och patientsäker vård.

Syfte
Att undersöka om personal och patienter/brukare inom vård och omsorg i Stockholms län har pågående eller genomgången SARS-CoV-2 infektion, som en kunskapsbas för att förstå hur infektionen sprids inom vård och omsorg och för att kunna planera en god och säker vård som både skyddar utsatta riskgrupper för smitta och bidrar till att tillse fullgod kapacitet inom vård och omsorg. Kunskapsunderlaget torde ge förutsättningar att beräkna kapacitetsbehovet för respektive vårdnivåer med avsevärt förbättrad precision.

Studieupplägg och metodologi
Personal och patienter/brukare inom vård och omsorg i Stockholms län tillfrågas om att delta i studien med skriftlig forskningspersonsinformation, varefter skriftligt samtycke inhämtas. Studien utgår från Karolinska Universitetssjukhuset, men personal och patienter/brukare även vid annan vård och omsorg i länet kommer att tillfrågas om de vill delta. För de forskningspersoner som samtycker tas ett prov från näsan (nasofarynx) för påvisning och sekvensbestämning av SARS-CoV-2 med kvantitativ PCR samt för sekvenering. Genomgången SARS-CoV-2 infektion undersöks genom att mäta förekomsten av SARS-CoV-2 antikroppar i serum med hjälp av de metoder som idag finns tillgängliga, Luminex (Immunoassay), Immunofluoresence och ELISA. För detta tas vid samma provtagningstillfälle ett blodprov (serumrör) om 5 milliliter.

Studiepopulation
Personal samt patienter/brukare som inte har en konstaterad, eller misstänkt, SARS-CoV-2 infektion inom vård och omsorg i Stockholms län.

Kriterier
Inklusionskriterier (alla tre uppfyllda):

- anställd eller patient/brukare inom vård och omsorg i Stockholms län
- Mellan 18-99 år
- Lämnat samtycke att delta i studien

Exklusionskriterier:

- de som inte lämnat informerat samtycke till att delta i studien
- de med redan bekräftad SARS-CoV-2 infektion
- de som redan provtagits för SARS-CoV-2 infektion på grund av klinisk misstanke, men där svar ej kommit än.

Studieperiod
Studien kommer att starta så fort som möjligt, helst 14 april 2020 och pågå under 2 veckor.

Studieobjekt
Personal och patienter/brukare på de olika verksamheterna kommer att tillfrågas att delta i studien av en utsedd medicinskt ansvarig kontaktperson. De avdelningar som har personal som är mest exponerad för smitta (intensivvård, infektion och akuten) samt de avdelningar som har särskilt sköra patienter (i synnerhet geriatrik) tillfrågas först .

Forskningsplan

Studiebeskrivning
Provtagning utförs av utbildad personal, företrädesvis i tillfälliga provtagningslokaler uppsatta utanför respektive vårdinrättning. Lokalerna och provtagande personal är utrustade med adekvat skyddsutrustning. Näsprover (nasofarynxprover) analyseras för förekomst av SARS-CoV-2 med PCR samt sekveneras vid Karolinska Universitetslaboratoriet. Serumprover analyseras med de vid tillfället bästa tillgängliga antikroppstesterna (preliminärt med Luminex (Immunoassay), Immunofluoresence och ELISA). Serumanalyser utförs företrädesvis vid Karolinska Universitetslaboratoriet (KUL). Sammanställning och utsvar görs av medicinskt ansvarig personal på KUL. Forskningspersonerna delges provsvar och dess tolkning via provsvarsmodulen inom 1177 Vårdguiden (1177 PEP - patientens egen provtagning). Vid positivt svar (förekomst av SARS-CoV-2) delges uppgiften medicinskt ansvarig vid verksamheten för handläggning enligt vid tidpunkten gällande riktlinjer från Smittskydd Stockholm (dessa kan ändras över tid men har fram till nu varit isolering i 14 dagar eller vid allvarligare symptom uppsöka vård).

Datainsamling
Antikroppstitrar mot Corona uppmäts på uppdrag av KUL och sammanställs vid ME Klinisk Immunologi, KUL. Corona-PCR och sekvenering utförs också under KULs ansvar.
En samlad databas innehållande namn, personnummer, vårdavdelning, provtagningsdatum samt resultat av alla Corona-analyser upprättas vid Karolinska Universitetslaboratoriet, där den förvaras under skalskydd och vårdsekretess.
Karolinska Universitetssjukhusets redan befintliga administrativa databaser innehåller uppgift om när och var medarbetarna varit i tjänst och deras adresser. Vidare finns uppgift om när patienter skrivits in, för vilka diagnoser och vilka medicinska åtgärder som vidtagits. Motsvarande databaser finns på alla akutsjukhusen.

Dataanalys
Huvudsakliga utfallsmått är förekomst av SARS-CoV-2 (pågående infektion) samt förekomst av antikroppar mot SARS-CoV-2 (mått på genomgången infektion).
För både pågående infektion och för genomgången infektion analyseras vilka faktorer som i främsta hand är associerade med infektionen. För medarbetare:
-Ålder
-Kön
-Folkbokföringsadress
-Arbetsplats
-Provtagningsdatum (smittspridningen går nu så fort att vi förväntar oss att även ett par dagars skillnad kommer att ha betydelse).
-Arbetsuppgifter
För patienter/brukare:
-Inskrivningsdatum
-Diagnos
-Vilka medicinska åtgärder som utförts

Analyserna planeras utföras med multivariat logistisk regression med Relativ Risk som utfallsmått.

Data från sekvensbestämning av viruset planeras att jämföras mellan olika positiva prov för att undersöka om det rör sig om samma virus-isolat. Virusets arvsmassa förändras snabbt över tid och om arvsmassan har ungefärligen samma sekvens tyder det på samma smittkälla. Sådan information kan vara till mycket stor nytta för att förstå hur och i vilka sammanhang viruset sprids.

Data från analysen av virusantikroppar planeras att utföras uppdelat på om låga, medel eller höga nivåer av antikroppar förekommer, med samma analys för eventuell association med de faktorer som är listade ovan. Det är väsentligt att förstå vilka faktorer som kan påverka immunsvaret mot viruset.

Reglering

Informerat samtycke inhämtas skriftligen från forskningspersonerna i samband med provtagningen. Originalansökan har etiskt tillstånd från Etikprövningsmyndigheten (EPM) (2020-01620).

Referenser

WHO, Laboratory testing for coronavirus disease (COVID-19) in suspected human cases: interim guidance, 19 march 2020. WHO reference number: WHO/COVID-19/laboratory/2020.5.

Forskningsplan

Studier av pågående och genomgången SARS-CoV-2 infektion (som orsakar COVID-19) på akutsjukhus i Stockholms län

Etikprövningsbeslut

(pågående)

Medverkande forskare

Kalle Conneryd Lundgren, Operativ direktör, Karolinska Universitetssjukhuset

Joakim Dillner, FoU-Chef, Professor, Karolinska Universitetslaboratoriet, Karolinska Universitetssjukhuset

Lars Engstrand, Professor, Karolinska Universitetslaboratoriet, Karolinska Universitetssjukhuset

Lars I Eriksson, FoU-Chef, Professor, ME Intensivvård, Karolinska Universitetssjukhuset

Studiefakta

Typ av studie	Observationsstudie
Studiedesign	Kohortundersökning<br>Öppen studie
Inkludering ålder	18-99
Inkludering kön	Män och Kvinnor

Beskrivning

Bakgrund
En global pandemi av SARS-CoV-2 infektion orsakar COVID-19 sjukdom med hög dödlighet över hela världen. Vi har mycket bristande kännedom om hur infektionen sprids i sjukvården och det är helt nödvändig kunskap för att kunna planera en god och patientsäker vård.

Syfte
Att undersöka om personal och patienter vid Stockholms akutsjukhus har pågående eller genomgången SARS-CoV-2 infektion, som en kunskapsbas för att förstå hur infektionen sprids i sjukvården och för att kunna planera en god och säker vård som både skyddar utsatta riskgrupper för smitta och bidrar till att tillse fullgod kapacitet i sjukvården. Kunskapsunderlaget torde ge förutsättningar att beräkna kapacitetsbehovet för respektive vårdnivåer med avsevärt förbättrad precision.

Studieupplägg och metodologi
Personal och patienter vid akutsjukhus i Stockholms län tillfrågas om att delta i studien med skriftlig forskningspersonsinformation, varefter skriftligt samtycke inhämtas. Studien utgår från Karolinska Universitetssjukhuset, men övriga akutsjukhus i länet kommer att tillfrågas om de vill delta. För de forskningspersoner som samtycker tas ett prov från näsan (nasofarynx) för påvisning och sekvensbestämning av SARS-CoV-2, med kvantitativ PCR samt sekvenering. Genomgången SARS-CoV-2 infektion undersöks genom att mäta förekomsten av SARS-CoV-2 antikroppar i serum med hjälp av de metoder som idag finns tillgängliga, Luminex (Immunoassay), Immunofluoresence och ELISA. För detta tas vid samma provtagningstillfälle ett blodprov om 5 milliliter.

Forskningsplan

Studiepopulation
Personal samt patienter inlagda för annat än konstaterad, eller misstänkt, SARS-CoV-2 infektion vid Stockholms akutsjukhus.

Kriterier
Inklusionskriterier (alla tre uppfyllda):
- Inlagd eller anställd vid ett akutsjukhus i Stockholms län
- Mellan 18-99 år
- Lämnat samtycke att delta i studien

Exklusionskriterier:
- de som inte lämnat informerat samtycke till att delta i studien
- de med redan bekräftad SARS-CoV-2 infektion
- de som redan provtagits för SARS-CoV-2 infektion på grund av klinisk misstanke, men där svar ej kommit än.

Studieperiod
Studien kommer att starta så fort som möjligt, helst 6 april 2020 och pågå under 2 veckor.

Studieobjekt
Sjukhuspersonal och patienter på de olika avdelningarna kommer att tillfrågas att delta i studien av en av respektive sjukhus utsedd kontaktperson. De avdelningar som har personal som är mest exponerad för smitta (intensivvård, infektion och akuten) samt de avdelningar som har särskilt sköra patienter (i synnerhet geriatrik) tillfrågas först.

Studiebeskrivning
Provtagning utförs av utbildad personal i tillfälliga provtagningslokaler uppsatta utanför respektive sjukhus. Lokalerna och provtagande personal är utrustade med adekvat skyddsutrustning. Näsprover (nasofarynxprover) analyseras för förekomst av SARS-CoV-2 med PCR vid Karolinska Universitetslaboratoriet. Serumprover analyseras med de vid tillfället bästa tillgängliga antikroppstesterna (preliminärt med Luminex (Immunoassay), Immunofluoresence och ELISA). Serumanalyser utförs företrädesvis vid Karolinska Universitetslaboratoriet (KUL). Sammanställning och utsvar görs av medicinskt ansvarig personal på KUL. Forskningspersonerna delges provsvar och dess tolkning via provsvarsmodulen inom 1177 Vårdguiden (1177 PEP - patientens egen provtagning). Vid positivt svar (förekomst av SARS-CoV-2) delges uppgiften ansvarig Smittskyddsläkare för handläggning enligt vid tidpunkten gällande rekommendationer (dessa kan ändras över tid men har fram till nu varit isolering i 14 dagar eller vid allvarligare symptom uppsöka vård).

Datainsamling
Antikroppstitrar mot Corona uppmäts och sammanställs vid ME Klinisk Immunologi, KUL och Corona-PCR och sekvenering utförs också under KULs ansvar.
En samlad databas innehållande namn, personnummer, vårdavdelning, provtagningsdatum samt resultat av alla Corona-analyser upprättas vid Karolinska Universitetslaboratoriet, där den förvaras under skalskydd och vårdsekretess.
Karolinska Universitetssjukhusets redan befintliga administrativa databaser innehåller uppgift om när och var medarbetarna varit i tjänst och deras adresser. Vidare finns uppgift om när patienter skrivits

in, för vilka diagnoser och vilka medicinska åtgärder som vidtagits. Motsvarande databaser finns på alla akutsjukhusen.

Dataanalys
Huvudsakliga utfallsmått är förekomst av SARS-CoV-2 (pågående infektion) samt förekomst av antikroppar mot SARS-CoV-2 (mått på genomgången infektion).
För både pågående infektion och för genomgången infektion analyseras vilka faktorer som i främsta hand är associerade med infektionen. För medarbetare:
-Ålder
-Kön
-Folkbokföringsadress
-Arbetsplats
-Provtagningsdatum (smittspridningen går nu så fort att vi förväntar oss att även ett par dagars skillnad kommer att ha betydelse).
-Arbetsuppgifter
För patienter:
-Inskrivningsdatum
-Diagnos
-Vilka medicinska åtgärder som utförts

Analyserna planeras utföras med multivariat logistisk regression med Relativ Risk som utfallsmått.

Data från sekvensbestämning av viruset planeras att jämföras mellan olika positiva prov för att undersöka om det rör sig om samma virus-isolat. Virusets arvsmassa förändras snabbt över tid och om arvsmassan har ungefärligen samma sekvens tyder det på samma smittkälla. Sådan information kan vara till mycket stor nytta för att förstå hur och i vilka sammanhang viruset sprids.

Data från analysen av virusantikroppar planeras att utföras uppdelat på om låga, medel eller höga nivåer av antikroppar förekommer, med samma analys för eventuell association med de faktorer som är listade ovan. Det är väsentligt att förstå vilka faktorer som kan påverka immunsvaret mot viruset.

Personer med höga nivåer av antikroppar kommer även att tillfrågas om de vill donera blod för en separat studie av om plasma från tillfrisknade personer har effekt som behandling vid svår COVID-19 sjukdom (Studieplan, forskningspersonsinformation och bilaga 1 för EPM-ansökan för denna andra studie (Dnr. 2020-01479) medsändes som bilaga till denna ansökan).

Reglering
Informerat samtycke inhämtas skriftligen från forskningspersonerna i samband med provtagningen. Studien söker om etiskt tillstånd från Etikprövningsmyndigheten (EPM).

Referenser
WHO, Laboratory testing for coronavirus disease (COVID-19) in suspected human cases: interim guidance, 19 march 2020. WHO reference number: WHO/COVID-19/laboratory/2020.5.

Studier av pågående och genomgången SARS-CoV-2 infektion (som orsakar COVID-19) på akutsjukhus i Stockholms län.

Information till forskningsperson

Vi vill fråga dig om du vill delta i ett forskningsprojekt. I det här dokumentet får du information om projektet och om vad det innebär att delta.

Vad är det för projekt och varför vill ni att jag ska delta?

Du tillfrågas härmed om du vill delta i en studie där vi undersöker om medarbetare vid akutsjukhus och inskrivna patienter har pågående eller genomgången SARS-CoV-2 infektion. Vi vill förstå hur infektionen sprids i sjukvården för att kunna planera en god och säker vård. Personal och patienter vid akutsjukhus i Stockholms län får erbjudande att delta i studien. Deltagandet i studien är helt frivilligt. Ansvarig för studien är Karolinska Universitetssjukhuset.

Hur går studien till?

Ett näsprov tas genom att en mjuk, tunn pinne förs in i näsan några sekunder. Därefter tas ett blodprov (5ml) i armbågsvecket. Proverna skickas på analys för förekomst av coronaviruset i näsprovet och förekomst av antikroppar mot coronaviruset i blodet. Du får tillgång till dina provsvar genom att logga in på dina sidor i 1177 Vårdguiden. Vården kommer även att kontakta dig om det skulle visa sig att du bär på coronaviruset.

Möjliga följder och risker med att delta i studien

Att ta näsprov kan upplevas som obehagligt och ibland något smärtsamt. Vid blodprovstagningen kan viss smärta och blodutgjutning vid insticksstället förekomma.

Vad händer med mina uppgifter?

Projektet kommer att registrera information om dig.

Den information som samlas in under denna forskningsstudie kommer att behandlas av forskare vid Karolinska Universitetssjukhuset. Personuppgifter från studien kommer att lagras i en databas. Ändamålet med denna databas är i första hand forskning, i andra hand klinisk behandling. Dina uppgifter är sekretesskyddade och ingen obehörig har tillgång till databasen. Dina svar och resultat kommer att behandlas så att inte obehöriga kan ta del av dem. Vid databearbetning, då studien rapporteras eller publiceras kommer en enskild individ inte att kunna urskiljas. Studien följer gällande lagstiftning enligt EU:s dataskyddsförordning (GDPR) och Patientdatalagen (2008:355). Personuppgiftsansvarig myndighet är Region Stockholm, Box 22550, 104 22 Stockholm.

Du har rätt att skriftligen begära att få reda på vilka uppgifter som finns registrerade om dig. Ett sådant utdrag har du rätt att få en gång per år utan kostnad. Framkommer det att det står någonting felaktigt om dig ska den felaktiga uppgiften ändras. Om du önskar ett utdrag kontakta ansvarig forskare (se nedan).

Hur får jag information om resultatet av studien?

Resultatet av studien kommer att publiceras på internet, i vetenskapliga tidskrifter och presenteras i samband med vetenskapliga möten. Enbart statistik kommer att presenteras och ingen enskild person kommer att kunna identifieras. Du kan, men behöver inte, ta del av dina individuella data. Dina provsvar och tolkningen av dem erhåller du via 1177 Vårdguiden. Om dina provresultat visar att du har coronaviruset kommer vården att kontakta dig.

Försäkring och ersättning

Du har samma försäkringar mot skador som kan uppstå i denna studie som vid all sjukdomsbehandling i allmän vård genom Patientförsäkringen och Läkemedelsförsäkringen. Ingen extra ersättning utgår i studien då det inte kommer att medföra några extra kostnader för dig.

Deltagandet är frivilligt

Ditt deltagande är frivilligt och du kan när som helst välja att avbryta deltagandet. Om du väljer att inte delta eller vill avbryta ditt deltagande behöver du inte uppge varför, och det kommer inte heller att påverka din framtida vård eller behandling.

Om du vill avbryta ditt deltagande ska du kontakta den ansvariga för studien (se nedan).

Ansvariga för studien

Ansvarig för studien är Karolinska Universitetssjukhuset (Kontaktperson Joakim Dillner, tel.0724682460, e-post joakim.dillner@sll.se).

Samtycke till att delta i studien

Jag har fått muntlig och skriftlig informationen om studien och har haft möjlighet att ställa frågor. Jag får behålla den skriftliga informationen.

☐ Jag samtycker till att delta i ”Studier av pågående och genomgången SARS-CoV-2 infektion (som orsakar COVID-19) på akutsjukhus i Stockholms län.”

☐ Jag samtycker till att uppgifter om mig behandlas på det sätt som beskrivs i forskningspersonsinformationen.

Plats och datum	Underskrift

Curriculum Vitae, Joakim Dillner

Karolinska University Laboratory, Karolinska University Hsopital

141 83 Stockholm, Sweden;
e-mail: joakim.dillner@sll.se

University degree
1995: M.D. (Doctor of Medicine), Karolinska Institutet, Stockholm, Sweden.
Doctoral degree
1986: Dr. Med. Sc. in Tumor Biology, Karolinska Institutet, Stockholm, Sweden.

Relevant post-doctoral experience
1988-1992: Research Assistant, Dept of Virology, Karolinska Institutet, Stockholm, Sweden.
1986-1988: Postdoctoral Research Fellow, Scripps Clinic, La Jolla, California, USA.

Associate professorship "Docent"
1990: Docent in Virology, Karolinska Institutet, Stockholm, Sweden.

Present employment
2009-present: Professor in Infectious Disease Epidemiology. Karolinska Institutet, Stockholm, Sweden.
2017-present: Director of Research & Development (FoU-chef) at the Karolinska University Laboratory, Karolinska University Hospital.
2014-present: Head of Unit, Cervical Cancer Prevention Center, Karolinska University Hospital Laboratory, Dept. of Clinical Pathology & Cytology.
2012-present: Director of the Swedish National Quality Registry for Cervical Cancer Prevention (www.NKCx.se).

Previous employments and time of appointments
2001-2010: Professor of Virology, particularly Molecular Epidemiology. Dept. of Medical Microbiology, Lund University, Malmö, Sweden.
2001-2012: Clinical appointment in the Department of Clinical Microbiology, Region Skåne,
1996-1999: Visiting Professor, Dept of Infectious Disease Epidemiology, Finnish National Public Health Institute, Helsinki, Finland, with teaching responsibility at the Postgraduate School of Epidemiology, Tampere University School of Public Health, Tampere, Finland. Visiting professorship funded by the Nordic Academy for Advanced Studies.
1993-1997: Six-year position as Cancer Researcher at the Microbiology and Tumor Biology Center, Karolinska Institutet
1988-1992: Research Assistant at the Dept of Virology, Karolinska Institutet. Four-year position awarded by the Swedish Medical Research Council.
1986-1988: Postdoctoral Research Fellow at Scripps Clinic, La Jolla, California, USA.

Supervisor of Graduate Students and Postdoctoral Research Fellows
Main supervisor for 21 students with completed thesis: Lena Dillner, M.D. Ph.D, 1992. Pirkko Heino, Ph.D, 1996. Zhaohui Wang, Ph.D, 1999.Veronika af Geijersstam, Ph.D, 2000. Lennart Kjellberg, M.D., Ph.D, 2000. Kristina Elfgren, M.D, Ph.D. 2003. Xiaohong Wang, Ph.D, 2004. Lisen Arnheim, Ph.D, 2005. Kristina Hazard, Ph.D, 2007. Pontus Nauclér, Ph.D,

2007. Annika Lundstig, Ph.D, 2007. Malin Sjöholm, Ph.D, 2008. Janka Ryding, Ph, D, 2008. Zoltan Korodi, Ph.D, 2008. Anna Söderlund Strand, Ph.D, 2008. Kristin Andersson, Ph.D, 2010. Johanna Ekström, Ph.D, 2011. Sophia Harlid, Ph.D, 2011. Helena Faust, Ph. D, 2012, Davit Bzhalava, Ph.D, 2014, Sara Arroyo Muhr, Ph.D, 2016.
Main supervisor for 4 registered graduate students, ongoing: Maria Hortlund, Zurab Bzhalava, Hanna Artemchuk, Pontus Bjerre.
Post-doctoral Research Fellows in my group, ongoing (4): Karin Sundström, Miriam Elfström, Sara Arroyo Muhr, Emilie Hultin.
Previous supervision of postdoctoral research fellows: Davit Bzhalava, Vitaly Smelov, Tapio Luostarinen, Jannie Lam, Linda Eliasson, Ilvars Silins.

Scientific Production
500 Web of Science-indexed publications in the areas of cancer screening, vaccination, tumor virology, epidemiology and research infrastructures. The H-index is 74 with >24000 citations.

Major ongoing research programs & research infrastructures managed:
-Director of the Swedish National Cervical Screening Registry (www.nkcx.se), one of the largest National Quality Registries in Sweden that involves active collaboration with all units performing cervical cancer preventive work in Sweden.
-PI of the Strategic Research Foundation research program Biobank & Registry-based evaluation/Implementation of new Gynecological Health Technologies (BRIGHT) with the long-term goal to tailor screening intensity to each individual according to her biomarker determined risk of disease.
-Karolinska Institutet Core Facility in eHealth. Karolinska Institutet Core Facility grant for 2018-2021 awarded by the strategic research area healthcare sciences.

Forskningsplan

Plasma från personer som tillfrisknat från SARS-CoV-2 Coronainfektion som behandling vid akut COVID-19-sjukdom

Etikprövningsbeslut

(pågående)

Medverkande forskare

Joakim Dillner, Professor, Karolinska Universitetslabroatoriet/Karolinska Institutet

Maria Matl, Verksamhetschef, Transfusionsmedicn, Karolinska Universitetslaboratoriet

Anders Sönnerborg, Professor, Karolinska Universitetssjukhuset/Karolinska Institutet

Soo Aleman, Docent, ME Infektionssjukdomar. Karolinska Universitetssjukhuset

Jonas Klingström, Forskare, Centrum för infektionsmedicin, Inst f Medicin, Karolinska Institutet

Studiefakta

Typ av studie	Interventionsstudie
Studiedesign	Före-efter behandlingsinförande<br>Öppen studie<br>Primärt syfte: behandling
Inkludering ålder	18-80
Inkludering kön	Män och Kvinnor
Inkludering av friska frivilliga	Nej

Beskrivning

Bakgrund
Att ge plasma eller immunglobulinfraktionen från tillfriskande personer har använts med gott resultat för ett ganska stort antal infektioner, bl.a. parvovirus, cytomegalovirus, respiratory syncytical (RS) virus och Junin virus. WHO har i sina rekommendationer för hur en influensa-pandemi bör hanteras angivit att plasma från tillfrisknade ”may play a role” om det inte finns något annat att tillgå.

Syfte
Att undersöka säkerhet och effekt av om plasma från personer som tillfrisknat från en Coronavirus-infektion ges till personer med akut COVID-19 sjukdom.

Studieupplägg och metodologi
Före-Efter design, där det kliniska förloppet his dem som fått konvalescentplasma jämförs med det som kunnat uppmätas hos motsvarande patienter som lagts in innan behandlingen fanns att tillgå och kunnat erbjudas.

Studiepopulation
Patienter akut sjuka i COVID-19 vid Karolinska Universitetssjukhuset.

Kriterier
Inklusionskriterier (alla fyra uppfyllda):

Forskningsplan

- Inlagd vid Karolinska Universitetssjukhuset
- Konfirmerad Covid19 infektion SARS-CoV-2 med PCR
- Mellan 18-80 år
- Lämnat samtycke att delta i studien

Exklusionskriterier:

- ingen matchande plasmadonator (AB0 systemet)
- de som inte lämnat informerat samtycke till att delta i studien

Studieperiod

Studien kommer att starta så fort som möjligt, preferentiellt 1 april 2020, och pågå under 6 månader.

Studieobjekt

I en första fas för att i första hand studera säkerhet: ett litet antal (10) patienter med allvarlig Coronavirusinfektion som vårdas inom slutenvård.
I en andra fas 20 patienter som läggs in för slutenvård med svår Coronavirusinfektion.

Studiebeskrivning

Patienter som tillfrisknat från SARS-CoV-2 infektion och som behandlats vid Karolinska Universitetssjukhuset kommer att tillfrågas för plasmadonation och samtycken inhämtas vid positivt svar. Plasmagivare matchas mot patienter med blodgruppering enligt AB0-systemet. Förekomst av IgG antikroppar mot Coronavirus testas genom immunfluorescens mot Corona-virus-infekterade celler. Plasma från patienter med höga titrar används i första hand.
Plasmadonationerna genomförs inom sjukhusets ordinarie blodverksamhet och plasman testas för blodsmitta samt även för Corona (för att utesluta att det skulle kunna finnas kvar infektiöst virus i givarens blod). Plasma ges sedan först i mycket liten mängd till 10 patienter inom slutenvården (till en början med 1 ml) samtidigt som patienten observeras och beredskap för att hantera allergiska reaktioner finns tillhands. Dosen ökas sedan varje halvtimme (i steg, 5, 10, 50, 100ml) för att slutligen uppnå en normal transfusionsmängd (200ml). De första 10 patienterna monitoreras för alla kliniska tecken på eventuell biverkan som feber, huvudvärk, svimning eller allergisk reaktion. Blodstatus, med särskild uppmärksamhet på markörer för njurfunktion, följs 2 gånger samma dag som dosen gavs och sedan en gång varje dag fram till utskrivning. Om dessa 10 patienter kunnat transfunderas utan svår biverkan, provas behandlingen på 20 patienter som läggs in för slutenvård med svår Coronavirusinfektion. De följs upp med mätning av mängd virus, behov av syrgas samt tid till utskrivning eller överföring till intensivvård. Om det verkar finnas en effekt kan en sedvanlig randomiserad forskningsstudie planeras.

Datainsamling

Kliniska data om patienterna och deras sjukdomsförlopp insamlas vid ME Infektionssjukdomar. Antikroppstitrar mot Corona uppmäts vid Folkhälsomyndigheten. Blodgruppering enligt AB0 systemet utförs vid Transfusionsmedicin KUL, som även beställer testning för blodsmittor samt Corona-PCR från Klinisk Mikrobiologi, KUL. En samlad databas upprättas vid Karolinska Universitetslaboratoriet där den förvaras under skalskydd och vårdsekretess.

Dataanalys

Huvudsakliga utfallsmått är vårdtider innan utskrivning samt andelen patienter som kunnat skrivas ut friska. Dessa jämförs mellan de patienter som ingår i studien och hur det kliniska utfallet varit för COVID-19 patienter innan studien startades. För säkerhetsdelen loggas alla potentiella biverkningar under observationstiden.

Forskningsplan

Reglering

Informerat samtycke inhämtas skriftligen från forskningspersonerna och från plasmadonatorerna innan behandling/plasmagivning.

Ansökan om etiskt tillstånd för studien pågår.

Referenser

Luke, T. C. et al. Hark back: Passive immunotherapy for influenza and other serious Infections. *Crit Care Med*. 38. e66-e73. 2010.

Casadevall, A., Pirofski, L-A. The convalescent sera option for containing COVID-19. *J Clin Invest.* 2020. https://doi.org/10.1172/JCI138003.

Information till forskningsperson

Vi vill fråga dig om du vill delta i ett forskningsprojekt. I det här dokumentet får du information om projektet och om vad det innebär att delta.

Vad är det för projekt och varför vill ni att jag ska delta?

Du tillfrågas härmed om du vill delta i en studie där vi undersöker säkerhet och effekt av om plasma från personer som tillfrisknat från en Coronavirus-infektion ges till personer med akut COVID-19 sjukdom. Patienter som behandlas för svår coronainfektion vid Karolinska Universitetssjukhuset får erbjudande att delta i studien. Deltagandet i studien är helt frivilligt. Ansvarig för studien är Karolinska Universitetssjukhuset.

Hur går studien till?

Plasma från en givare ges intravenöst först i mycket liten mängd (i början med 1 ml). Dosen ökas sedan varje halvtimme för att efter 3 timmar uppnå en normal transfusionsmängd (200ml plasma). Behandlingen sker vid ett tillfälle och sker samtidigt som du får ordinarie behandling på den avdelning du är inlagd. Du observeras under hela behandlingen och beredskap finns för att hantera eventuella allergiska reaktioner.

Möjliga följder och risker med att delta i studien

Plasmatransfusion är en välbeprövad klinisk rutin vid ett flertal olika sjukdomstillstånd, men den är obeprövad som behandling mot Covid-19 och kan vara förenlig med risker som inte kan förutses. Eventuella biverkningar som kan förekomma är feber, huvudvärk, svimning eller allergisk reaktion. I sällsynta fall kan en plasmatransfusion leda till komplikationer i form av septisk chock, TRALI (Transfusionsrelaterad akut lungskada) eller TACO (Transfusion Associated Circulatory Overload = Övervätskning). Vid ett flertal olika infektioner är det effektivt mot infektionen att ge plasma från tillfrisknade, men det finns också exempel på infektioner där metoden inte har effekt eller till och med kan förvärra infektionen.

Vad händer med mina uppgifter?

Projektet kommer att registrera information om dig.

Den information som samlas in under denna forskningsstudie kommer att behandlas av forskare i Region Stockholm. Personuppgifter från studien kommer att lagras i en databas. Ändamålet med denna databas är i första hand forskning, i andra hand klinisk behandling. Dina uppgifter är sekretesskyddade och ingen obehörig har tillgång till databasen. Dina svar och resultat kommer att behandlas så att inte obehöriga kan ta del av dem. Vid databearbetning, då studien rapporteras eller publiceras kommer en enskild individ inte att kunna urskiljas. Studien följer gällande lagstiftning enligt EU:s dataskyddsförordning (GDPR) och Patientdatalagen (2008:355). Personuppgiftsansvarig myndighet är Region Stockholm, Box 22550, 104 22 Stockholm.

Du har rätt att skriftligen begära att få reda på vilka uppgifter som finns registrerade om dig. Ett sådant utdrag har du rätt att få en gång per år utan kostnad. Framkommer det att det står någonting felaktigt om dig ska den felaktiga uppgiften ändras. Om du önskar ett utdrag kontakta ansvarig forskare (se nedan).

Hur får jag information om resultatet av studien?

Resultatet av studien kommer att publiceras på internet, i vetenskapliga tidskrifter och presenteras i samband med vetenskapliga möten. Enbart statistik kommer att presenteras och ingen enskild person kommer att kunna identifieras. Du kan, men behöver inte, ta del av dina individuella data. Kontakta isåfall ansvarig forskare (se nedan).

Försäkring och ersättning

Du har samma försäkringar mot skador som kan uppstå i denna studie som vid all sjukdomsbehandling i allmän vård genom Patientförsäkringen och Läkemedelsförsäkringen. Ingen extra ersättning utgår i studien då det inte kommer att medföra några extra kostnader för dig.

Deltagandet är frivilligt

Ditt deltagande är frivilligt och du kan när som helst välja att avbryta deltagandet. Om du väljer att inte delta eller vill avbryta ditt deltagande behöver du inte uppge varför, och det kommer inte heller att påverka din framtida vård eller behandling.

Om du vill avbryta ditt deltagande ska du kontakta den ansvariga för studien (se nedan).

Ansvariga för studien

Ansvarig för studien är Karolinska Universitetssjukhuset (Kontaktperson professor Joakim Dillner tel. 0724682460, e-post Joakim.Dillner@sll.se).

Samtycke till att delta i studien

Jag har fått muntlig och skriftlig informationen om studien och har haft möjlighet att ställa frågor. Jag får behålla den skriftliga informationen.

☐ Jag samtycker till att delta i studien Plasma från personer som tillfrisknat från SARS-CoV-2 Coronainfektion som behandling vid akut COVID-19-sjukdom.

☐ Jag samtycker till att uppgifter om mig behandlas på det sätt som beskrivs i forskningspersonsinformationen.

Plats och datum	Underskrift

Information till forskningsperson

Vi vill fråga dig om du vill delta i ett forskningsprojekt. I det här dokumentet får du information om projektet och om vad det innebär att delta.

Vad är det för projekt och varför vill ni att jag ska delta?

Du tillfrågas härmed om du vill delta i en studie där du donerar plasma som sedan avses ges till patienter som är akut sjuka i COVID-19. Målet med studien är att utvärdera om plasma från tillfrisknade har effekt mot COVID-19 sjukdomen och om det är säkert när det ges till personer som är akut sjuka. Personer som har haft Corona infektion och tillfrisknat blir tillfrågade om att delta i studien genom att donera plasma. Deltagandet i studien är helt frivilligt. Ansvarig för studien är Karolinska Universitetssjukhuset.

Hur går studien till?

Innan plasmagivningen får du först göra en hälsodeklaration vilket innebär att svara på frågor om ditt hälsotillstånd. Vid plasmagivningen tappas blod via en venkanyl i ena armen för att leda ut blodet som sedan delas upp i blodkroppar och plasma i ett sterilt separationssystem. I en andra venkanyl i andra armen får du tillbaka dina röda blodkroppar och förlorar på så sätt inget järn. Du ger drygt en halv liter plasma vid ett tillfälle och det tar cirka 40 minuter. Hela processen är automatisk och sker med hjälp av en maskin vid en blodcentral.

Ditt blod kommer att testas för om det innehåller blodsmitta (HIV eller hepatitvirus) samt om det innehåller Coronavirus och antikroppar mot Coronavirus. Om blodet vid testning visar sig innehålla virus så kommer du att få information om det.

Möjliga följder och risker med att delta i studien

Plasmagivning sker enligt klinisk rutin och är en välbeprövad metod. Eventuella biverkningar som kan förekomma är obehag från stickstället, yrsel eller stickning i armar och ben under givningen orsakat av citrat som ges för att blodet inte ska koagulera..

Vad händer med mina uppgifter?

Projektet kommer att samla in och registrera information om dig.

Den information som samlas in under denna forskningsstudie kommer att behandlas av forskare i Region Stockholm. Personuppgifter från studien kommer att lagras i en databas. Ändamålet med denna databas är i första hand forskning, i andra hand klinisk behandling. Dina uppgifter är sekretesskyddade och ingen obehörig har tillgång till databasen. Dina svar och resultat kommer att behandlas så att inte obehöriga kan ta del av dem. Vid databearbetning, då studien rapporteras eller publiceras kommer en enskild individ inte att kunna urskiljas. Studien följer gällande lagstiftning enligt EU:s dataskyddsförordning (GDPR) och Patientdatalagen (2008:355). Personuppgiftsansvarig myndighet är Region Stockholm, Box 22550, 104 22 Stockholm.

Du har rätt att skriftligen begära att få reda på vilka uppgifter som finns registrerade om dig. Ett sådant utdrag har du rätt att få en gång per år utan kostnad. Framkommer det att det står någonting felaktigt om dig ska den felaktiga uppgiften ändras. Om du önskar ett utdrag kontakta ansvarig forskare (se nedan).

Hur får jag information om resultatet av studien?

Resultatet av studien kommer att publiceras på internet, i vetenskapliga tidskrifter och presenteras i samband med vetenskapliga möten. Enbart statistik kommer att presenteras och ingen enskild person kommer att kunna identifieras. Om ditt blod vid testning visar sig innehålla virus så kommer vi att ge dig information om det.

Försäkring och ersättning

Du har samma försäkringar mot skador som kan uppstå i denna studie som vid all sjukdomsbehandling i allmän vård genom Patientförsäkringen och Läkemedelsförsäkringen. Ingen extra ersättning utgår i studien då det inte kommer att medföra några extra kostnader för dig.

Deltagandet är frivilligt

Ditt deltagande är frivilligt och du kan när som helst välja att avbryta deltagandet. Om du väljer att inte delta eller vill avbryta ditt deltagande behöver du inte uppge varför, och det kommer inte heller att påverka din framtida vård eller behandling.

Om du vill avbryta ditt deltagande ska du kontakta den ansvariga för studien (se nedan).

Ansvariga för studien

Ansvarig för studien är Karolinska Universitetssjukhuset (Kontaktperson professor Joakim Dillner tel. 0724682460, e-post Joakim.Dillner@sll.se).

Samtycke till att delta i studien

Jag har fått muntlig och skriftlig informationen om studien och har haft möjlighet att ställa frågor. Jag får behålla den skriftliga informationen.

☐ Jag samtycker till att delta i studien Plasma från personer som tillfrisknat från SARS-CoV-2 Coronainfektion som behandling vid akut COVID-19-sjukdom.

☐ Jag samtycker till att uppgifter om mig behandlas på det sätt som beskrivs i forskningspersonsinformationen.

Plats och datum	Underskrift

Ansökan om etikprövning – *Bilaga 1*
Beskrivning av forskningsprojektet

2. Typ av forskning

2.1. På vilket eller vilka sätt handlar projektet om forskning enligt 3-4 §§ etikprövningslagen?

- [x] 3 § 1 Forskningen kommer att samla in känsliga personuppgifter.
- [ ] 3 § 2 Forskningen kommer att samla in personuppgifter om lagöverträdelser.
- [x] 4 § 1 Forskningen innebär ett fysiskt ingrepp på en forskningsperson.
- [x] 4 § 2 Forskningen utförs enligt en metod som syftar till att påverka forskningspersonen fysiskt eller psykiskt, eller så innebär forskningen en uppenbar risk att skada forskningspersonen.
- [x] 4 § 3 Forskningen avser studier på biologiskt material som har tagits från en levande människa och kan härledas tillbaka till denna människa.
- [ ] 4 § 4 Forskningen avser ett fysiskt ingrepp på en avliden människa.
- [ ] 4 § 5 Forskningen avser studier på biologiskt material som tagits från en avliden människa och kan härledas tillbaka till denna människa.

- [ ] Forskningen faller inte under etikprövningslagens tillämpningsområde.

Forskningsperson: De levande människor som forskningen avser.

Personuppgifter: All slags information som direkt eller indirekt kan hänföras till en fysisk person som är i livet.

Känsliga personuppgifter: Känsliga personuppgifter är uppgifter som avslöjar ras eller etniskt ursprung, politiska åsikter, religiös eller filosofisk övertygelse, medlemskap i fackförening, hälsa, en persons sexualliv eller sexuella läggning, genetiska uppgifter och biometriska uppgifter som entydigt identifierar en person.

Personuppgifter om lagöverträdelser: Personuppgifter om lagöverträdelser som innefattar brott, domar i brottmål, straffprocessuella tvångsmedel eller administrativa frihetsberövanden.

2.2. [Om 3 § 1] **Ange vilken typ av känsliga personuppgifter som kommer behandlas i projektet.**

- [ ] ras eller etniskt ursprung
- [ ] politiska åsikter
- [ ] religiös eller filosofisk övertygelse
- [ ] medlemskap i fackförening
- [x] hälsa
- [ ] en persons sexualliv eller sexuella läggning
- [ ] genetiska uppgifter
- [ ] biometriska uppgifter som entydigt identifierar en person.

Personuppgifter: All slags information som direkt eller indirekt kan hänföras till en fysisk person som är i livet.

Känsliga personuppgifter: Känsliga personuppgifter är uppgifter som avslöjar ras eller etniskt ursprung, politiska åsikter, religiös eller filosofisk övertygelse, medlemskap i fackförening, hälsa, en persons sexualliv eller sexuella läggning, genetiska uppgifter och biometriska uppgifter som entydigt identifierar en person. Enligt definition i EU:s Dataskyddsförordning.

OBS! Om inga känsliga personuppgifter kommer behandlas i projektet så behöver frågan inte besvaras.

3. Syfte och frågeställningar

3.1. Skriv en populärvetenskaplig sammanfattning av forskningsprojektet (max 300 ord).

Beskriv projektet på ett sammanfattande vis. Tänk på att texten ska kunna förstås av personer som inte har vetenskaplig kompetens. Undvik därför terminologi som kräver specialkunskaper.

Plasma från tillfrisknade personer har använts för behandling av personer med svår infektionssjukdom ända sedan 1918 under den s.k. spanska sjukan. Dödligheten befanns halveras. Detta var långt innan upptäckten av antikroppar – idag vet vi att det fungerar genom att man överför skyddande (neutraliserande) antikroppar som bildas efter genomgången infektion.
Biverkningar som tidigare kunde uppstå innan upptäckten av blodgrupper och innan upptäckten av hur man testar för blodsmitta kan förhindras med dagens metoder och kunskap. Dessuom finns en omfattande klinisk erfarenhet av metoden.
Att ge plasma eller immunglobulinfraktionen från tillfriskande personer har använts med gott resultat för ett ganska stort antal infektioner, bl.a. parvovirus, cytomegalovirus, respiratory syncytical (RS) virus och Junin virus. WHO har i sina rekommendationer för hur en influensa-pandemi bör hanteras angivit att plasma från tillfrisknade ”may play a role” under en akut fas då det inte finns andra behandlingar att tillgå. Det finns således både en tydlig vetenskaplig bas för behandlingen, samt goda resultat från ett flertal virussjukdomar.
Vi vill prova med att insamla plasma från personer som tillfrisknat från Coronavirusinfektion för att kunna ge den till patienter med svår Coronavirus-sjukdom. För just Corona är det inte tydligt känt om metoden provats förut, men kinesiska internetsidor anger att metoden provats på 254 COVID-19 patienter med gott resultat. Amerikanska FDA upmanar till insamling av plasma från tillfrisknade så att detta skall finnas att tillgå.

3.2. Vad är det vetenskapliga syftet med projektet?

Beskriv det övergripande syftet med projektet. Redogör för vad det är för forskningsproblem som projektet ska behandla och vilka avgränsningar som gjorts.

Att insamla plasma från personer som tillfrisknat från Coronavirusinfektion med SARS-CoV-2 för att kunna ge den till patienter svårt sjuka i COVID-19. Hypotesen är att detta kommer att bidra till ett lindrigare sjukdomsförlopp. Den sannolika mekanismen är att neutralisera infektionen med antikroppar som bildats efter genomgången infektion. Studien begränsas initialt till 10 patienter inom slutenvården med allvarlig COVID-19 (säkerhet). Om behandlingen befinns vara säker avser vi att prova behandlingen på ytterligare 20 patienter i liknande tillstånd för att uppmäta om en effekt kan ses. Det kliniska förloppet jämförs sedan med det som kunnat uppmätas hos motsvarande patienter som lagts in innan behandlingen fanns att tillgå och kunnat erbjudas (utvärdering med s.k. före-efter design).
Vi har i detta läge inte tillräcklig information för att utforma en randomiserad forskningsstudie. Information om säkerhet och en ungefärlig uppfattning om hur stor effekt som kan förväntas krävs både för att etiskt avgöra nytta-vinst samt för att beräkna en sådan studies storlek.

3.3. Vilka är de vetenskapliga frågeställningarna?

Ange klart och tydligt den eller de vetenskapliga frågeställningarna i projektet.

Är det säkert att ge plasma från personer som tillfrisknat från en SARS-CoV-2 infektion till akut sjuka coronainfekterade patienter? Kan en trolig klinisk effekt av en sådan behandling uppmätas?

4. Metod

4.1. Redogör för metod inkl. proceduren, tekniken eller behandlingen.

Det ska framgå hur projektet planeras att genomföras. Beskriv insamlade datas karaktär och ange hur datas tillförlitlighet ska säkerställas.

Om enkäter och intervjuer ingår ska tillvägagångssätt, frågornas innehåll och hur slutsatser dras beskriva. Bifoga enkäter och skattningsskalor.

För medicinsk forskning ska anges t.ex. typer av ingrepp, mätmetoder, antal besök, tidsåtgång, doser, och administrationssätt för eventuella läkemedel.

Om projektet är ett samarbete med utlandet ska det tydligt framgå vilken del av forskningen som utförs i Sverige och vilken del av forskningen som utförs utanför Sverige. Det är endast forskning som ska utföras i Sverige som Etikprövningsmyndigheten kan pröva.

Patienter som tillfrisknat från SARS-CoV-2 infektion (COVID-19) efter att ha diagnostiserats vid Karolinska Universitetssjukhuset tillfrågas om de vill donera plasma för forskningsändamål och skriftligt samtycke inhämtas. Plasmagivare matchas mot akut sjuka COVID-19-patienter med blodgruppering enligt AB0-systemet. Plasmadonationerna genomförs inom sjukhusets ordinarie blodverksamhet och plasman testas för blodsmittor med PCR-metodik samt även för Corona med PCR (för att utesluta att det skulle kunna finnas kvar infektiöst virus i givarens blod). Plasma ges sedan först i mycket liten mängd till patienter inom slutenvården (början med 1 ml) samtidigt som patienten observeras och beredskap för att hantera allergiska reaktioner finns tillhands. Dosen ökas sedan varje halvtimme (stegvis till 5, 10, 50, 100ml) för att slutligen uppnå en normal transfusionsmängd (200ml). Patienterna bevakas kontinuerligt under beredskap för eventuella akuta eller fördröjda komplikationer av immunologisk- eller icke-immunologisk karaktär.

4.2. Redogör för på vilket sätt metoden skiljer sig från klinisk rutin eller den ordinarie behandlingen.

Ange vad som avviker eller tillkommer med anledning av forskningsprojektet.

Patienterna behandlas i övrigt helt enligt klinisk rutin. Plasmatransfusion är också en klinisk rutin, men inte på denna indikation.

4.3. Redogör för tidigare erfarenheter (egna och/eller andras) av den använda proceduren, tekniken eller behandlingen.

Redogör för vilken erfarenhet och kompetens som medverkande forskare har av att använda den procedur, teknik eller behandling om man planerar att använda i projektet. Redogör även för vilken erfarenhet som finns generellt eller globalt.

Att ge plasma eller immunglobulinfraktionen från tillfriskande personer har använts med gott resultat för ett ganska stort antal infektioner, bl.a. parvovirus, cytomegalovirus, respiratory syncytical (RS) virus och Junin virus. WHO har i sina rekommendationer för hur en influensa-pandemi bör hanteras angivit att plasma från tillfrisknade ”may play a role” i ett akutskede då det inte finns andra behandlingar att tillgå. Att ge plasma är en rutinmetod med omfattande klinisk dokumentation. Det ges t.ex. vid omfattande blödningar eller oklara koagulationsrubbningar. Det finns detaljerade anvisningar om både kontraindikationer samt åtgärder för att minimera risker. Transfusionsmedicin vid Karolinska Universitetslaboratoriet är ett ledande transfusionsmedicinskt centrum i Sverige.

5. Tidsplan

5.1. Förväntat startdatum för projektet:

Ange om möjligt, beräknad start av projektet.

2020-04-01 eller det datum då tillstånd ges.

5.2. Förväntat slutdatum för projektet:

Ange om möjligt, beräknat slut av projektet.

2022-12-31

5.3. Tidsplan för de olika delar som ingår i projektet:

Pilotstudie med 10 patienter förväntas genomföras under första månaden av projektet. Efter utvärdering och om förväntat resultat erhålls, genomförs en effektstudie med 20 patienter med historiskt jämförelsematerial. Dessa delar av studien kan ligga till grund för ställningstagande till och evt. design av senare randomiserade studier (säkerhet samt ungefärlig storlek på effekt måste vara känd för att kunna planera randomiserade studier). Den kliniska delen av studien förväntas vara klar under de närmsta månaderna, men analys av studieresultat kan ta längre tid.

Redogör för projektets tidsschema, hur de olika delarna förhåller sig till varandra, vid behov kan ett förklarande flödesschema bifogas.

6. Datainsamling

6.1. Redogör för datainsamling och datas karaktär.

Redogör för hur datainsamlingen ska gå till. Beskriv den data som ska samlas in och hur den ska samlas in.

All data samlas inom befintliga dokumentationssystem i vården.

6.2. Redogör för det statistiska underlaget för studiepopulationen/ undersökningsmaterialets storlek.

Redogör för de beräkningar och överväganden som gjorts för att komma fram till hur många deltagare som behövs för att uppnå tillräcklig statistisk styrka. Redogörelsen av hur man kommit fram till lämpligt urval ska beskriva projektets möjligheter att besvara frågeställningarna.

Pilotstudien med 10+20 patienter är en kvalitativ studie där säkerhet och det kliniska förloppet hos behandlade patienter jämförs med det som kunnat uppmätas hos motsvarande patienter som lagts in innan behandlingen fanns att tillgå och kunnat erbjudas. Beroende av utfallet från denna pilotstudie kan en randomiserad studie på ett större antal deltagare planeras. Denna studie är också avhängig av hur SARS-CoV-2 epidemin utvecklar sig.

6.3. Hur kommer undersökningsprocedurerna att dokumenteras?

Redogör för hur undersökningarna och eventuella ingrepp dokumenteras. Ange om band- och videoinspelningar kommer användas.

Undersökningarna och behandlingarna dokumenteras med sedvanlig vårddokumentation.

6.4. Hur kommer insamlad data att hanteras och förvaras?

Redogör för hur data kommer att hanteras efter insamlingen. Om data kommer att pseudonymiseras (”kodas”) ska kodningsförfarandet beskrivas. Det ska framgå var kodlistorna/kodnycklarna kommer att förvaras samt vem/vilka som kommer ha tillgång till dem. Det ska även framgå hur länge data kommer att sparas samt om det kommer att avidentifieras (genom att kodnyckel förstörs) eller förstöras i sin helhet.

All datahantering och dokumentation sker inom sjukvårdens datasystem och under vårdsekretess.

7. Etiska överväganden

7.1. Vilka risker kan ett deltagande medföra för de forskningspersoner som ingår i forskningsprojektet?

Risker kan vara av olika karaktär. Det kan exempelvis vara fysisk eller psykisk skada, smärta, obehag, integritetsintrång på kort eller lång sikt. Ange vilka risker det kan finnas för de forskningspersoner som deltar i detta forskningsprojekt.

Plasmadonatorerna kan tänkas känna det svårt att tacka nej. Det finns idag endast 16 tillfrisknade personer i Sverige, samtidigt som antalet akut sjuka ökar exponentiellt. Enbart donatorer som fyller kriterierna för blodgivning kommer att godkännas för plasmagivning. För donatorerna finns också samma risker som vid all blodgivning, som smärta och blodutgjutning vid insticksstället samt trötthet efter blodgivning. Det kan också tänkas finnas integritetsrisker. All information och tappning vid besök på blodcentral sker dock under vårdsekretess.
För mottagarna kan det inte uteslutas att plasma från tillfrisknade skulle kunna innebära särskilda risker vid just Corona. Det är t.ex. tänkbart att det skulle kunna bildas immunkomplex mellan antikroppar och virus som skulle kunna ge allergiska reaktioner eller deponeras i njurarna med njurskador som följd. Riskerna för mottagarna kan vara antingen akuta (ex cirkulatorisk överbelastning, lungskada, anafylaktisk reaktion) eller fördröjda (immunmodulation, hemolys). Det finns också beskrivet att antikroppar med dåligt skydd mot viruset kan ge ett förvärrat klinisk förlopp om de "tränger ut" kroppens egen produktion av skyddande antikroppar. Det är därför viktigt att endast samla plasma från personer som verkligen blivit friska.

7.2. Vilken nytta kan ett deltagande medföra för de forskningspersoner som ingår i forskningsprojektet?

Ange vilken hjälp de forskningspersoner som deltar i detta forskningsprojekt kan få som ett resultat av forskningsprojektet.

Om behandlingen befinns vara säker och ha effekt så får forskningspersonerna en mildare COVID-19 sjukdom.

7.3. Gör en värdering av förhållandet mellan riskerna och nyttan av projektet.

Forskning får enligt etikprövningslagen bara godkännas om riskerna som den kan medföra för forskningspersonernas hälsa, säkerhet och personliga integritet uppvägs av dess vetenskapliga värde.

Den, för Corona, obeprövade behandlingen avses provas på akut sjuka patienter med COVID-19 där inga etablerade och effektiva behandlingar finns att tillgå. Givande av plasma från tillfrisknade har provats vid i princip samtliga vid akuta epidemier och har i flertalet fall varit effektivt Plasmatransfusion är en välbeprövad behandling mot andra sjukdomstillstånd och risken för att forskningspersonernas tillstånd skulle förvärras av behandlingen bedöms som liten. I skrivande stund har 7 patienter i Region Stockholm avlidit till följd av COVID-19. Nyttan bedöms därför överväga de risker som forskningspersonerna i studien utsätts för.

7.4. Beskriv hur projektet har utformats för att minimera riskerna för forskningspersonerna.

Forskning får enligt etikprövningslagen bara godkännas om det förväntade resultatet inte kan nås på ett annat sätt som innebär mindre risker för forskningspersoners hälsa, säkerhet och personliga integritet.

Behandling av personuppgifter som avses i 3 § får godkännas bara om den är nödvändig för att forskningen ska kunna utföras.

Observera att om en behandling ska prövas för första gången på människa måste detta tydligt framgå och relevanta säkerhetsrutiner tydligt beskrivas.

Ange vilka risker som ett deltagande i forskningsprojektet kan innebära. Finns det risker t.ex. med att ingå i en kontrollgrupp?

För att minimera riskerna för forskningspersonerna inleds projektet med säkerhetsstudier av ett litet antal (10) patienter med akut Coronavirusinfektion som vårdas inom slutenvård. Plasma ges först i mycket liten mängd (början med 1 ml) samtidigt som patienten observeras och beredskap för att hantera allergiska reaktioner finns tillhands. Dosen ökas sedan varje halvtimme (i steg, 5, 10, 50, 100 ml) för att slutligen uppnå en normal transfusionsmängd (200ml). Forskningspersonerna monitoreras för alla kliniska tecken på eventuell biverkan som feber, huvudvärk, svimning eller allergisk reaktion. Blodstatus, med särskild uppmärksamhet på markörer för njurfunktion, följs 2 gånger samma dag som dosen gavs och sedan en gång varje dag fram till utskrivning.

7.5. Identifiera och precisera om eventuella etiska problem (nackdelar/fördelar) kan uppstå i ett vidare perspektiv genom forskningsprojektet.

Redovisa t.ex. om vissa grupper (andra än de forskningspersoner som ingår i forskningsprojektet) kan komma att utpekas respektive få hjälp som ett resultat av projektet. Frågan avser även de indirekta riskerna eller den indirekta nyttan. T.ex. genetisk påverkan på kommande generationer eller om resultaten på annat sätt kan tänkas skada vissa grupper.

Om behandlingen är framgångsrik och epidemin ökar exponentiellt kan det bli ett etiskt problem att den enda behandlingen är beroende av ett litet antal donatorer och att behandlingen inte kan ges till alla som behöver den.

8. Forskningspersoner

8.1. Hur görs urvalet av forskningspersoner?

Forskningsperson: De levande människor som forskningen avser.

Beskriv vilka forskningspersoner som kommer inkluderas i projektet. Redogör för de överväganden som gjorts vid valet av forskningspersoner. Om vissa grupper utesluts från deltagande i projektet ska en motivering till uteslutandet framgå.

Redogör även för hur forskaren kommer i kontakt med eller får kännedom om lämpliga forskningspersoner.

Forskningspersonerna utgörs i första steget (blodgivning) av de personer som diagnostiserats med COVID-19 vid Karolinska Sjukhuset men som förklarats friska. I ett andra steg (mottagare av plasma) tillfrågas patienter som skrivits in vid slutenvården vid Karolinska Universitetssjukhuset till följd av en SARS-CoV-2 infektion (COVID-19). Patienterna identifieras av de för vården medicinskt ansvariga vid infektionskliniken på Karolinska Universitetssjukhuet. Vi avser att endast inkludera beslutskompetenta som kan ge samtycke.

8.2. Hur många forskningspersoner kommer att inkluderas i forskningsprojektet?

Ange hur många forskningspersoner som totalt kommer inkluderas i projektet samt i förekommande fall hur många som kommer inkluderas i olika delprojekt.

Initialt 10+20 forskningspersoner enligt bifogad forskningsplan.

8.3. Vilka urvalskriterier kommer att användas för inklusion?

Ange vilka kriterier som måste uppfyllas för att en forskningsperson ska inkluderas i projektet.

Urvalskriterier för inklusion är: samtliga patienter inom slutenvården vid Karolinska Universitetssjukhuset i Huddinge som är akut sjuka i COVID-19 och ger samtyucke till att delta, till dess att en kohort på 10+20 forskningspersoner (som har matchande blodgrupp till plasmadonatorerna)uppnåtts.

8.4. Vilka urvalskriterier kommer att användas för exklusion?

Ange vilka kriterier som måste uppfyllas för att en forskningsperson ska exkluderas ur projektet.

Urvalskriterier för exklusion är: Avsaknad av samtycke. Forskningspersoner till vilka inga matchande plasmadonatorer kan erhållas.

8.5. Ange relationen mellan forskare och forskningspersonerna.

Ange forskarens roll i relation till forskningspersonens roll. Det kan t.ex. vara som behandlare (läkare, psykolog, fysioterapeut etc.) och patient/klient, som lärare och student eller arbetsgivare och anställd. All form av relation som kan tänkas medföra risk för påverkan ska beskrivas. Enligt etikprövningslagen ska information och samtycke ägnas särskild uppmärksamhet om forskningspersonen står i ett beroendeförhållande till huvudmannen, forskaren eller antas ha svårigheter att ta tillvara sin rätt.

Det finns ingen relation mellan studiens forskare och forskningspersonerna.

8.6. Vilket försäkringsskydd finns för de forskningspersoner som deltar i forskningsprojektet?

Forskningshuvudmannen har ansvar för att kontrollera att det finns försäkring som täcker eventuella skador som kan uppkomma i samband med forskningen. Ange vilka försäkringar som forskningspersonen kommer att omfattas av samt vilket skydd försäkringarna ger forskningspersonen.

Projektet genomförs inom Region Stockholms verksamhet, där sedvanlig patientförsäkring gäller.

8.7. Redogör för den beredskap som finns för att hantera oväntade bifynd eller händelser under forskningsprocessen som kan äventyra forskningspersonernas säkerhet.

Beskriv vilken tillgång forskningsprojektet har till utrustning, personal och kompetens för att hantera eventuellt oväntade komplikationer eller bifynd. Beskriv även vilken planering som finns för att hantera eventuella oväntade komplikationer eller bifynd.

Vid oväntade komplikationer för forskningspersonerna står Karolinska Universitetssjukhusets kompetens inom intensivvård till förfogande.

8.8. Kommer ekonomisk ersättning eller andra förmåner betalas ut till forskningspersonerna?

Forskningspersonerna kan utöver ersättning för resor, förlorad arbetsinkomst eller andra utgifter erhålla viss ersättning för obehag och besvär. Ersättningen ska vara skälig. Om barn eller ungdomar under 18 år deltar i forskningsprojektet får sådan ersättning inte vara stor och den bör inte erbjudas i samband med rekryteringen. Vid klinisk läkemedelsprövning med barn eller ungdomar under 18 år får inga incitament eller ekonomiska förmåner ges, undantaget kostnadsersättningar.

Nej

8.8.1. [Om Ja 8.8] Vilken ekonomisk ersättning kommer betalas ut och när?

9. Information och samtycke

9.1. Kommer forskningspersonerna att informeras om forskningsprojektet och tillfrågas om de vill vara med eller inte?

Grundregeln, enligt etikprövningslagen, är att forskning bara utförs om forskningspersonen har informerats och samtyckt till deltagande. Information till forskningspersonerna kan ges både muntligt och skriftligt.

Ja

9.1.1. [Om Ja 9.1] Hur, när (i vilket skede) och av vem informeras och tillfrågas forskningspersonerna?

Beskriv proceduren för hur information ges och samtycke inhämtas. Vem som frågar, när detta sker och hur samtycket dokumenteras. På vilket sätt säkerställs att forskningspersonen ges betänketid och möjlighet att ställa frågor.

Utförlig redovisning är särskilt viktig när det ingår barn eller personer med nedsatt beslutskompetens i forskningsprojektet.

Ansvarig läkare vid Medicinsk Enhet Infektionssjukdomar kontaktar de från COVID-19 tillfrisknade och tillfrågar dem om de är villiga att ge blod. Dessa ges skriftlig information och kan endast ge blod efter samtycke. Forskningspersonerna för mottagande av plasma kommer att informeras och tillfrågas av behandlande läkare vid Medicinsk Enhet Infektionssjukdomar. Plasmagivningen genomförs när läkare vid Bloddonation godkänner givaren. Patienten ges en detaljerad skriftlig information om behandlingen där det tydligt framgår att den är oprövad för Corona samt att den kan vara förenlig med risker som vi inte kan förutse. Vi avser att kräva skriftligt samtycke.

9.1.2. [Om Nej 9.1] Motivera varför forskningspersonerna inte ska informeras och tillfrågas.

Lämna en utförlig redogörelse för de avvägningar som har gjorts och de skäl som ligger till grund för bedömningen att forskningspersonerna inte ska informeras och tillfrågas. Observera att forskning utan information och samtycke endast är möjlig i undantag fall och i den forskning sam avses i 3 § etikprövningslagen, eller 20–22 § i etikprövningslagen.

Max 4500 tecken

9.2. Kommer barn under 18 år att ingå i forskningsprojektet?

För barn under 15 år måste barnets samtliga vårdnadshavare ge sin tillåtelse (samtycka) till att barnet får delta i forskningsprojektet.

Barn mellan 15 och 18 år, som inser vad forskningen innebär för hans eller hennes del, ska informeras om och lämna eget samtycke till forskningen. I andra fall ska barnets samtliga vårdnadshavare ge sin tillåtelse (samtycka) till att barnet får delta i forskningsprojektet.

Forskningen får, trots vårdnadshavarnas tillåtelse (samtycke) inte utföras om barnet motsätter sig att forskningen utförs.

Nej

9.2.1. [Om Ja 9.2] Ange barnens ålder.

9.3. Kommer forskningspersoner, vars mening på grund av sjukdom, psykisk störning, försvagat hälsotillstånd eller något annat liknande förhållande inte kan inhämtas, att ingå i forskningsprojektet?

Ange om forskningsprojektet kommer involvera personer som själva inte kan samtycka till sitt eget deltagande p.g.a. sjukdom, psykisk störning, försvagat hälsotillstånd eller liknande tillstånd.

Nej

9.3.1. [Om Ja 9.3] **Motivera varför denna grupp av forskningspersoner ska ingå i projektet.**

Forskning utan samtycke på denna grupp av forskningspersoner får endast utföras om forskningen förväntas ge en kunskap som inte är möjlig att få genom forskning med samtycke.

Forskningen ska dessutom förväntas leda till direkt nytta för forskningspersonen. Alternativt ska forskningen bidra till ett resultat som kan vara till nytta för forskningspersonen eller annan som lider av samma eller liknande sjukdom eller tillstånd samt innebära en obetydlig risk för skada eller obehag för forskningspersonen. Forskningspersonen ska så långt som möjligt informeras personligen om forskningen.

Samråd ska ske med närmaste anhörig. Samråd ska också ske med god man eller förvaltare om frågan ingår i uppdraget som god man eller förvaltare. Forskningen får inte utföras om forskningspersonen i någon form ger uttryck för att inte vilja delta eller om någon av dem som samråd har skett med motsätter sig införandet.

Om studien verkar kunna påvisa en effekt och det då inkommer kritiskt sjuka och medvetslösa patienter med COVID-19 så skulle det kunna tänkas uppstå en diskussion om att erbjuda plasmaferes. Det är svårt att säga om det skulle kunna inträffa.

9.3.2. [Om Ja 9.3] **Beskriv hur samråd med närmaste anhörig, god man eller förvaltare kommer att ske.**

Vid forskning på forskningspersoner, vars mening inte går att inhämta, ska samråd ske med närmaste anhörig som ska få möjlighet att motsätta sig deltagandet. Samråd ska ske med god man eller förvaltare om frågan ingår i uppdraget som god man eller förvaltare.

Vi avser att begränsa studien till beslutskompetenta forskningspersoner. Om anhöriga medföljer får de samma skriftliga information som patienten. Givande av plasma till medvetslösa är knappast lämpligt innan säkerhet och effekt etablerats, vilket vi avser göra med denna studie.

10. Registeruppgifter

10.1. Kommer projektet att begära ut uppgifter från ett befintligt register?

Här avses alla typer av register som innehåller personuppgifter eller uppgifter som tidigare varit personuppgifter men senare avidentifierats.

Nej

10.1.1. [Om Ja 9.1] Ur vilket eller vilka register kommer uppgifterna att begäras?

Namnge registret eller registren som uppgifterna kommer att begäras ifrån. Ange även huvudman för respektive register.

Max 2000 tecken

10.1.2. [Om Ja 9.1] Vilka uppgifter kommer att begäras ut och varför?

Beskriv vilken typ av uppgifter som kommer att begäras ut och varför de behövs för att besvara projektets frågeställningar.

En komplett variabellista kan med fördel bifogas som bilaga. Etikprövningsmyndigheten behöver inte alltid en redovisning på variabelnivå för att göra sin bedömning, men det kan ibland underlätta vid begäran hos registerhållaren om den kompletta variabellistan funnits med vid etikprövningen.

Max 4500 tecken

11. Resultat från djurförsök

11.1. Finns det relevanta resultat från djurförsök?

Frågan avser framförallt klinisk behandlingsforskning. Om djurförsök inte har utförts, ange då anledningen till detta.

Ej Aktuellt

11.1.1. [Om Ja 11.1] Redogör för resultaten av djurförsöken

Redogör övergripande för de djurförsök som utförts och vilka resultat de gav. Framförallt ska de resultat som är av relevans för detta forskningsprojekt redogöras för.

För ett flertal andra infektionssjukdomar finns det resultat från djurförsök, där metoden använts med god effekt.
För COVID-19 finns inga djurförsök. Det är en pandemi och relevanta djurmodeller saknas.

12. REDOVISNING AV RESULTAT

12.1. Hur garanteras tillgång till data för forskningshuvudmannen och medverkande forskare?

Normalt ska den som ansvarar för genomförandet av forskningen ha full tillgång till data. Om flera forskare samverkar i uppdragsforskning bör den forskare som är huvudansvarig för genomförandet i förväg komma överens med övriga forskare om tillgång till data.

Alla data hanteras av forskningshuvudmannen och medverkande forskare.

12.2. Vem eller vilka ansvarar för databearbetning och skriftlig redovisning av resultaten?

Ange vem eller vilka som kommer bearbeta och analysera forskningsdata och vem eller vilka som kommer utforma den skriftliga redovisningen. Normalt ska den som ansvarar för genomförandet av forskningen ha full tillgång till data.

Huvudansvarig forskare tillsammans med medverkande forskare ansvarar för databearbetning och skriftlig redovisning av resultaten.

12.3. Hur och när planeras resultaten att offentliggöras?

Ange i vilken form resultaten planeras att offentliggöras. Exempelvis vetenskaplig publicering med peer review, open acess, intern rapport. Ange om möjligt en tidsplan för offentliggörandet.

Huvudresultatet kommer att publiceras direkt på internet samt uppföljas med publicering i vetenskapliga artiklar och presenteras på konferenser samt kommuniceras till beslutsfattare i frågan

12.4. På vilket sätt garanteras forskningspersonernas rätt till integritet när materialet offentliggörs?

Redogör för hur data presenteras när den offentliggörs och hur forskningspersonernas integritet skyddas vid offentliggörandet.

Data redovisas enbart i aggregerad form, utan att kunna hänföras till enskild individ.

13. EKONOMISKA FÖRHÅLLANDEN

13.1. Redovisa eventuella ekonomiska överenskommelser med bidragsgivare eller andra finansiärer (namn och belopp).

Redovisa alla överenskommelser om finansiering som har slutits med den eller de som ska genomföra forskningen. Ange vilka belopp som kommer att erhållas för forskningsprojektet och vad ersättningen ska täcka. Ange även eventuella belopp per forskningsperson.

Studien genomförs inom ordinarie hälsovård.

13.2. Redovisa forskningshuvudmannens, huvudansvarig forskares och medverkande forskares egna ekonomiska intressen.

Redovisa egna ekonomiska intressen i form av t.ex. aktieinnehav, anställning, konsultuppdrag i finansierade företag, eget företag som kan få direkt eller indirekt vinst av forskningen.

Samtliga medverkande forskare har inga dylika intressen att redovisa för denna studie.

Studier av pågående och genomgången SARS-CoV-2 infektion (som orsakar COVID-19) inom vård och omsorg i Stockholms län.

Information till forskningsperson

Vi vill fråga dig om du vill delta i ett forskningsprojekt. I det här dokumentet får du information om projektet och om vad det innebär att delta.

Vad är det för projekt och varför vill ni att jag ska delta?

Du tillfrågas härmed om du vill delta i en studie där vi undersöker om omvårdnadspersonal har pågående eller genomgången SARS-CoV-2 infektion. Vi vill förstå hur infektionen sprids i omvårdnadsverksamheten för att kunna planera en god och säker omvårdnad. Personal, brukare och patienter inom vård och omsorg i Stockholms län får erbjudande att delta i studien. Deltagandet i studien är helt frivilligt. Ansvarig för studien är Karolinska Universitetssjukhuset.

Hur går studien till?

Ett svalgprov tas genom att en mjuk, tunn pinne förs in i svalget några sekunder. Därefter tas ett blodprov (5ml) i armbågsvecket. Proverna skickas på analys för förekomst av coronaviruset i svalgprovet och förekomst av antikroppar mot coronaviruset i blodet, som är ett tecken på genomgången infektion. När Ditt provsvar är klart får du ett SMS med en länk till en hemsida där du kan se ditt provsvar efter inloggning med Mobilt Bank-ID. Vården kommer även att kontakta dig om det skulle visa sig att du bär på coronaviruset.

Positiva svar på pågående COVID-19 måste enligt Smittskyddslag (2004:168) anmälas till smittskyddsläkaren och Folkhälsomyndigheten. Positiva svar innebär även att vårdpersonal behöver stanna hemma i 14 dagar och varit symptomfria i minst 48 timmar. Informationen delges arbetsgivaren.

Möjliga följder och risker med att delta i studien

Att ta svalgprov kan upplevas som obehagligt och ibland något smärtsamt. Vid blodprovstagningen kan viss smärta och blodutgjutning vid insticksstället förekomma.

Vad händer med mina uppgifter?

Projektet kommer att registrera information om du har pågående eller genomgången SARS-CoV-2 infektion. För att bättre förstå infektionens spridning kommer informationen att kombineras med information som redan finns i vårdgivarens administrativa databaser (Personuppgifter samt uppgifter om hälsa och när man varit i tjänst).

Den information som samlas in under denna forskningsstudie kommer att behandlas av forskare vid Karolinska Universitetssjukhuset. Personuppgifter från studien kommer att lagras i en databas hos sjukhusledningen. Ändamålet med denna databas är i första hand forskning, i andra hand klinisk behandling. Dina uppgifter är sekretesskyddade och ingen obehörig har tillgång till databasen. Dina svar och resultat kommer att behandlas så att inte obehöriga kan ta del av dem. Vid databearbetning, då studien rapporteras eller publiceras kommer en enskild individ inte att kunna urskiljas. Studien följer gällande lagstiftning enligt EU:s dataskyddsförordning (GDPR) och Patientdatalagen (2008:355). Personuppgiftsansvarig myndighet är Region Stockholm, Box 22550, 104 22 Stockholm. Du har rätt att skriftligen begära att få reda på vilka uppgifter som finns registrerade om dig. Ett sådant utdrag har du rätt att få en gång per år utan kostnad. Framkommer det att det står någonting felaktigt om dig ska den felaktiga uppgiften ändras. Om du önskar ett utdrag kontakta ansvarig forskare (se nedan). Du kan också begära att behandlingen av dina personuppgifter begränsas. Om du har synpunkter på hanteringen av data har du möjlighet att kontakta Karolinska Universitetssjukhusets dataskyddsombud (dataskyddsombud.karolinska@sll.se). I händelse av vidare klagomål kan du därefter vända dig till Datainspektionen, som är tillsynsmyndighet.

Studier av pågående och genomgången SARS-CoV-2 infektion (som orsakar COVID-19) inom vård och omsorg i Stockholms län.

Vad händer med mina prover?

De prover som tas i studien förvaras efter analys kodade i en så kallad biobank. Biobankens namn är Stockholms medicinska biobank reg.nr 914. Huvudman för biobanken är Region Stockholm. Du har rätt att säga nej till att proverna sparas. Om du samtycker till att proverna sparas du rätt att senare ta tillbaka (ångra) det samtycket. Dina prover kommer i så fall att kastas eller avidentifieras. Om du vill ångra ett samtycke ska du kontakta ansvarig forskare.

Proverna får bara användas på det sätt som du har gett samtycke till. Om det skulle tillkomma forskning som ännu inte är planerad, kommer etikprövningsnämnden att besluta om du ska tillfrågas på nytt. Prover kommer att förvaras kodade, vilket innebär att proven inte direkt kan härledas till dig som person. Proverna och den tillhörande kodnyckeln kommer att förvaras åtskilda från varandra och skyddas från åtkomst av obehöriga. Kodnyckeln förvaras hos Karolinska Universitetssjukhuset.

Analyser kommer att ske inom Sverige och på Karolinska Universitetssjukhusets uppdrag. Dina kodade prov kan komma att skickas för analys inom Sverige.

Hur får jag information om resultatet av studien?

Resultatet av studien kommer att publiceras på internet, i vetenskapliga tidskrifter och presenteras i samband med vetenskapliga möten. Enbart statistik kommer att presenteras och ingen enskild person kommer att kunna identifieras. Du kan, men behöver inte, ta del av dina individuella data. När Dina provsvar är klara får Du ett SMS om hur du kan ta del av dem och tolkningen av dem. Om Du saknar mobiltelefon, ange på samtyckesblanketten hur du vill ta del av provsvaret. Om dina provresultat visar att du har coronaviruset kommer vården att kontakta dig.

Försäkring och ersättning

Du har samma försäkringar mot skador som kan uppstå i denna studie som vid all sjukdomsbehandling i allmän vård genom Patientförsäkringen. Ingen ersättning utgår i studien då det inte kommer att medföra några extra kostnader för dig.

Deltagandet är frivilligt

Ditt deltagande är frivilligt och du kan när som helst välja att avbryta deltagandet. Om du väljer att inte delta eller vill avbryta ditt deltagande behöver du inte uppge varför, och det kommer inte heller att påverka din framtida vård eller behandling.

Om du vill avbryta ditt deltagande ska du kontakta den ansvariga för studien (se nedan).

Ansvariga för studien

Ansvarig för studien är Karolinska Universitetssjukhuset (Kontaktperson Joakim Dillner, tel.0724682460, e-post joakim.dillner@sll.se).

Samtycke till att delta i studien

Jag har fått skriftlig informationen om studien och har haft möjlighet att ställa frågor. Jag får behålla den skriftliga informationen.

☐ Jag samtycker till att delta i ”Studier av pågående och genomgången SARS-CoV-2 infektion (som orsakar COVID-19) inom vård och omsorg i Stockholms län.”

☐ Jag samtycker till att uppgifter om mig behandlas på det sätt som beskrivs i forskningspersonsinformationen.

☐ Jag samtycker till att prover sparas i en biobank på det sätt som beskrivs i forskningspersonsinformationen.

Underskrift forskningsperson	Namnförtydligande	Datum

Mobiltelefonnummer dit SMS om färdigt provsvar kan skickas	Personnummer

Jag bekräftar att jag gett skriftlig information om studien och forskningspersonen har getts tillfälle att ställa frågor. Prover är vederbörligen märkta och forskningspersonen har givit skriftligt samtycke inklusive personnummer och mobiltelefonnummer. Detta dokument är upprättat i två original, varav forskningspersonen behåller det ena och det andra inskickas tillsammansmed proven till Covid19-studien, Forskningsgatan F52, Karolinska Universitetssjukhuset i Huddinge där det arkiveras av ansvarig forskare.

Underskrift kliniken	Namnförtydligande	Datum

Provtagande klinik

Studier av pågående och genomgången SARS-CoV-2 infektion (som orsakar COVID-19) inom vård och omsorg i Stockholms län.

Information till forskningsperson

Vi vill fråga dig om du vill delta i ett forskningsprojekt. I det här dokumentet får du information om projektet och om vad det innebär att delta.

Vad är det för projekt och varför vill ni att jag ska delta?

Du tillfrågas härmed om du vill delta i en studie där vi undersöker om brukare av vård och omsorg i Stockholms län har pågående eller genomgången SARS-CoV-2 infektion. Vi vill förstå hur infektionen sprids i omsorgsverksamheten för att kunna planera en god och säker vård. Personal, brukare och patienter inom vård och omsorg i Stockholms län får erbjudande att delta i studien. Deltagandet i studien är helt frivilligt. Ansvarig för studien är Karolinska Universitetssjukhuset.

Hur går studien till?

Ett svalgprov tas genom att en mjuk, tunn pinne förs in i svalget några sekunder. Därefter tas ett blodprov (5ml) i armbågsvecket. Proverna skickas på analys för förekomst av coronaviruset i svalgprovet och förekomst av antikroppar mot coronaviruset i blodet, som är ett tecken på genomgången infektion. När Ditt provsvar är klart får du ett SMS med en länk till en hemsida där du kan se ditt provsvar efter inloggning med Mobilt Bank-ID. Vården kommer även att kontakta dig om det skulle visa sig att du bär på coronaviruset.

Positiva svar på pågående COVID-19 måste enligt Smittskyddslag (2004:168) anmälas till smittskyddsläkaren och Folkhälsomyndigheten. Positiva svar innebär även att vårdpersonal behöver stanna hemma i 14 dagar och varit symptomfria i minst 48 timmar. Informationen delges arbetsgivaren.

Möjliga följder och risker med att delta i studien

Att ta svalgprov kan upplevas som obehagligt och ibland något smärtsamt. Vid blodprovstagningen kan viss smärta och blodutgjutning vid insticksstället förekomma.

Vad händer med mina uppgifter?

Projektet kommer att registrera information om du har pågående eller genomgången SARS-CoV-2 infektion. För att bättre förstå infektionens spridning kommer att informationen att kombineras med information som redan finns i vårdgivarens administrativa databaser (Personuppgifter samt uppgifter om hälsa och när man varit i tjänst).

Den information som samlas in under denna forskningsstudie kommer att behandlas av forskare vid Karolinska Universitetssjukhuset. Personuppgifter från studien kommer att lagras i en databas hos sjukhusledningen. Ändamålet med denna databas är i första hand forskning, i andra hand klinisk behandling. Dina uppgifter är sekretesskyddade och ingen obehörig har tillgång till databasen. Dina svar och resultat kommer att behandlas så att inte obehöriga kan ta del av dem. Vid databearbetning, då studien rapporteras eller publiceras kommer en enskild individ inte att kunna urskiljas. Studien följer gällande lagstiftning enligt EU:s dataskyddsförordning (GDPR) och Patientdatalagen (2008:355). Personuppgiftsansvarig myndighet är Karolinska Universitetssjukhuset, Box 22550, 104 22 Stockholm. Du har rätt att skriftligen begära att få reda på vilka uppgifter som finns registrerade om dig. Ett sådant utdrag har du rätt att få en gång per år utan kostnad. Framkommer det att det står någonting felaktigt om dig ska den felaktiga uppgiften ändras. Om du önskar ett utdrag kontakta ansvarig forskare (se nedan). Du kan också begära att behandlingen av dina personuppgifter begränsas. Om du har synpunkter på hanteringen av data har du möjlighet att kontakta Karolinska Universitetssjukhusets dataskyddsombud (dataskyddsombud.karolinska@sll.se). I händelse av vidare klagomål kan du därefter vända dig till Datainspektionen, som är tillsynsmyndighet.

Studier av pågående och genomgången SARS-CoV-2 infektion (som orsakar COVID-19) inom vård och omsorg i Stockholms län.

Vad händer med mina prover?

De prover som tas i studien förvaras efter analys kodade i en så kallad biobank. Biobankens namn är Stockholms medicinska biobank reg.nr 914. Huvudman för biobanken är Region Stockholm. Du har rätt att säga nej till att proverna sparas. Om du samtycker till att proverna sparas har du rätt att senare ta tillbaka (ångra) det samtycket. Dina prover kommer i så fall att kastas eller avidentifieras. Om du vill ångra ett samtycke ska du kontakta ansvarig forskare.

Proverna får bara användas på det sätt som du har gett samtycke till. Om det skulle tillkomma forskning som ännu inte är planerad, kommer etikprövningsnämnden att besluta om du ska tillfrågas på nytt. Prover kommer att förvaras kodade, vilket innebär att proven inte direkt kan härledas till dig som person. Proverna och den tillhörande kodnyckeln kommer att förvaras åtskilda från varandra och skyddas från åtkomst av obehöriga. Kodnyckeln förvaras hos Karolinska Universitetssjukhuset.

Analyser kommer att ske inom Sverige och på Karolinska Universitetssjukhusets uppdrag. Dina kodade prov kan komma att skickas för analys inom Sverige.

Hur får jag information om resultatet av studien?

Resultatet av studien kommer att publiceras på internet, i vetenskapliga tidskrifter och presenteras i samband med vetenskapliga möten. Enbart statistik kommer att presenteras och ingen enskild person kommer att kunna identifieras. Du kan, men behöver inte, ta del av dina individuella data. När Dina provsvar är klara får Du ett SMS om hur du kan ta del av dem och tolkningen av dem. Om Du saknar mobiltelefon, ange på samtyckesblanketten hur du vill ta del av provsvaret. Om dina provresultat visar att du har coronaviruset kommer vården att kontakta dig.

Försäkring och ersättning

Du har samma försäkringar mot skador som kan uppstå i denna studie som vid all sjukdomsbehandling i allmän vård genom Patientförsäkringen. Ingen ersättning utgår i studien då det inte kommer att medföra några extra kostnader för dig.

Deltagandet är frivilligt

Ditt deltagande är frivilligt och du kan när som helst välja att avbryta deltagandet. Om du väljer att inte delta eller vill avbryta ditt deltagande behöver du inte uppge varför, och det kommer inte heller att påverka din framtida vård eller behandling.

Om du vill avbryta ditt deltagande ska du kontakta den ansvariga för studien (se nedan).

Ansvariga för studien

Ansvarig för studien är Karolinska Universitetssjukhuset (Kontaktperson Joakim Dillner, tel.0724682460, e-post joakim.dillner@sll.se).

Samtycke till att delta i studien

Jag har fått skriftlig informationen om studien och har haft möjlighet att ställa frågor. Jag får behålla den skriftliga informationen.

☐ Jag samtycker till att delta i ”Studier av pågående och genomgången SARS-CoV-2 infektion (som orsakar COVID-19) på akutsjukhusen i Stockholms län.”

☐ Jag samtycker till att uppgifter om mig behandlas på det sätt som beskrivs i forskningspersonsinformationen.

☐ Jag samtycker till att prover sparas i en biobank på det sätt som beskrivs i forskningspersonsinformationen.

Underskrift forskningsperson	Namnförtydligande	Datum

Mobiltelefonnummer dit SMS om färdigt provsvar kan skickas	Personnummer

Jag bekräftar att jag gett skriftlig information om studien och forskningspersonen har getts tillfälle att ställa frågor. Prover är vederbörligen märkta och forskningspersonen har givit skriftligt samtycke inklusive personnummer och mobiltelefonnummer. Detta dokument är upprättat i två original, varav forskningspersonen behåller det ena och det andra inskickas tillsammansmed proven till Covid19-studien, Forskningsgatan F52, Karolinska Universitetssjukhuset i Huddinge där det arkiveras av ansvarig forskare.

Underskrift kliniken	Namnförtydligande	Datum

Provtagande klinik

Studier av pågående och genomgången SARS-CoV-2 infektion (som orsakar COVID-19) på akutsjukhus i Stockholms län.

Information till forskningsperson

Vi vill fråga dig om du vill delta i ett forskningsprojekt. I det här dokumentet får du information om projektet och om vad det innebär att delta.

Vad är det för projekt och varför vill ni att jag ska delta?

Du tillfrågas härmed om du vill delta i en studie där vi undersöker om medarbetare vid akutsjukhus och inskrivna patienter har pågående eller genomgången SARS-CoV-2 infektion. Vi vill förstå hur infektionen sprids i sjukvården för att kunna planera en god och säker vård. Personal och patienter vid akutsjukhus i Stockholms län får erbjudande att delta i studien. Deltagandet i studien är helt frivilligt. Ansvarig för studien är Karolinska Universitetssjukhuset.

Hur går studien till?

Ett svalgprov tas genom att en mjuk, tunn pinne förs in i svalget några sekunder. Därefter tas ett blodprov (5ml) i armbågsvecket. Proverna skickas på analys för förekomst av coronaviruset i svalgprovet och förekomst av antikroppar mot coronaviruset i blodet som är ett tecken på genomgången infektion. När Ditt provsvar är klart får du ett SMS med en länk till en hemsida där du kan se ditt provsvar efter inloggning med Mobilt Bank-ID. Vården kommer även att kontakta dig om det skulle visa sig att du bär på coronaviruset.

Positiva svar på pågående COVID-19 måste enligt Smittskyddslag (2004:168) anmälas till smittskyddsläkaren och Folkhälsomyndigheten. Positiva svar innebär även att vårdpersonal behöver stanna hemma i 14 dagar och varit symptomfria i minst 48 timmar. Informationen delges arbetsgivaren.

Möjliga följder och risker med att delta i studien

Att ta svalgprov kan upplevas som obehagligt och ibland något smärtsamt. Vid blodprovstagningen kan viss smärta och blodutgjutning vid insticksstället förekomma.

Vad händer med mina uppgifter?

Projektet kommer att registrera information om du har pågående eller genomgången SARS-CoV-2 infektion. För att bättre förstå infektionens spridning kommer att informationen att kombineras med information som redan finns i vårdgivarens administrativa databaser (Personuppgifter samt uppgifter om hälsa och när man varit i tjänst).

Den information som samlas in under denna forskningsstudie kommer att behandlas av forskare vid Karolinska Universitetssjukhuset. Personuppgifter från studien kommer att lagras i en databas hos sjukhusledningen. Ändamålet med denna databas är i första hand forskning, i andra hand klinisk behandling. Dina uppgifter är sekretesskyddade och ingen obehörig har tillgång till databasen. Dina svar och resultat kommer att behandlas så att inte obehöriga kan ta del av dem. Vid databearbetning, då studien rapporteras eller publiceras kommer en enskild individ inte att kunna urskiljas. Studien följer gällande lagstiftning enligt EU:s dataskyddsförordning (GDPR) och Patientdatalagen (2008:355). Personuppgiftsansvarig myndighet är Region Stockholm, Box 22550, 104 22 Stockholm. Du har rätt att skriftligen begära att få reda på vilka uppgifter som finns registrerade om dig. Ett sådant utdrag har du rätt att få en gång per år utan kostnad. Framkommer det att det står någonting felaktigt om dig ska den felaktiga uppgiften ändras. Om du önskar ett utdrag kontakta ansvarig forskare (se nedan). Du kan också begära att behandlingen av dina personuppgifter begränsas. Om du har synpunkter på hanteringen av data har du möjlighet att kontakta Karolinska Universitetssjukhusets dataskyddsombud (dataskyddsombud.karolinska@sll.se). I händelse av vidare klagomål kan du därefter vända dig till Datainspektionen, som är tillsynsmyndighet.

Studier av pågående och genomgången SARS-CoV-2 infektion (som orsakar COVID-19) på akutsjukhus i Stockholms län.

Vad händer med mina prover?

De prover som tas i studien förvaras kodade i en så kallad biobank. Biobankens namn är Stockholms medicinska biobank reg.nr 914. Huvudman för biobanken är Region Stockholm. Du har rätt att säga nej till att proverna sparas. Om du samtycker till att proverna sparas har du rätt att senare ta tillbaka (ångra) det samtycket. Dina prover kommer i så fall att kastas eller avidentifieras. Om du vill ångra ett samtycke ska du kontakta ansvarig forskare.

Proverna får bara användas på det sätt som du har gett samtycke till. Om det skulle tillkomma forskning som ännu inte är planerad, kommer etikprövningsnämnden att besluta om du ska tillfrågas på nytt. Prover kommer att förvaras kodade, vilket innebär att proven inte direkt kan härledas till dig som person. Proverna och den tillhörande kodnyckeln kommer att förvaras åtskilda från varandra och skyddas från åtkomst av obehöriga. Kodnyckeln förvaras hos Karolinska Universitetssjukhuset.

Analyser kommer att ske inom Sverige och på Karolinska Universitetssjukhusets uppdrag. Dina kodade prov kan komma att skickas för analys inom Sverige.

Hur får jag information om resultatet av studien?

Resultatet av studien kommer att publiceras på internet, i vetenskapliga tidskrifter och presenteras i samband med vetenskapliga möten. Enbart statistik kommer att presenteras och ingen enskild person kommer att kunna identifieras. Du kan, men behöver inte, ta del av dina individuella data. När Dina provsvar är klara får Du ett SMS om hur du kan ta del av dem och tolkningen av dem. Om Du saknar mobiltelefon, ange på samtyckesblanketten hur du vill ta del av provsvaret. Om dina provresultat visar att du har coronaviruset kommer vården att kontakta dig.

Försäkring och ersättning

Du har samma försäkringar mot skador som kan uppstå i denna studie som vid all sjukdomsbehandling i allmän vård genom Patientförsäkringen. Ingen   ersättning utgår i studien då det inte kommer att medföra några extra kostnader för dig.

Deltagandet är frivilligt

Ditt deltagande är frivilligt och du kan när som helst välja att avbryta deltagandet. Om du väljer att inte delta eller vill avbryta ditt deltagande behöver du inte uppge varför, och det kommer inte heller att påverka din framtida vård eller behandling.

Om du vill avbryta ditt deltagande ska du kontakta den ansvariga för studien (se nedan).

Ansvariga för studien

Ansvarig för studien är Karolinska Universitetssjukhuset (Kontaktperson Joakim Dillner, tel.0724682460, e-post joakim.dillner@sll.se).

Samtycke till att delta i studien

Jag har fått skriftlig informationen om studien och har haft möjlighet att ställa frågor. Jag får behålla den skriftliga informationen.

☐ Jag samtycker till att delta i ”Studier av pågående och genomgången SARS-CoV-2 infektion (som orsakar COVID-19) på akutsjukhusen i Stockholms län.”

☐ Jag samtycker till att uppgifter om mig behandlas på det sätt som beskrivs i forskningspersonsinformationen.

☐ Jag samtycker till att prover sparas i en biobank på det sätt som beskrivs i forskningspersonsinformationen.

Underskrift forskningsperson	Namnförtydligande	Datum

Mobiltelefonnummer dit SMS om färdigt provsvar kan skickas	Personnummer

Jag bekräftar att jag gett skriftlig information om studien och forskningspersonen har getts tillfälle att ställa frågor. Prover är vederbörligen märkta och forskningspersonen har givit skriftligt samtycke inklusive personnummer och mobiltelefonnummer. Detta dokument är upprättat i två original, varav forskningspersonen behåller det ena och det andra inskickas tillsammansmed proven till Covid19-studien, Forskningsgatan F52, Karolinska Universitetssjukhuset i Huddinge där det arkiveras av ansvarig forskare.

Underskrift kliniken	Namnförtydligande	Datum

Provtagande klinik
