# Supplementary material for: Potential SARS-CoV-2 infectiousness among asymptomatic healthcare workers
Source: PLoS One. 2021 Dec 17;16(12):e0260453. doi: 10.1371/journal.pone.0260453 (PMC8682911; doi:10.1371/journal.pone.0260453)
Supplement: Attachment — Submitted filename: Response to Reviewers.docx [file pone.0260453.s002.docx]

Rebuttal letter for PONE-D-21-25144

**Please, see below our on-by-one responses to the reviewers comments:**

Reviewer #1:

The primary objective of a secondary study was to quantify the proportion of healthcare workers who were potentially infectious. Their sick leave was classified as post-symptomatic or pre-symptomatic, and virus-positive subjects with no sick leave were considered asymptomatic. About 0.2% of he HCW at work were potentially infectious and pre-symptomatic (later had disease) and 0.17% were potentially infectious and asymptomatic (never took sick leave). In summary, 33% and 28% of all the 57 potentially infectious subjects were pre-symptomatic or asymptomatic, respectively.

Major revisions:
1. Include a statistical analysis section which lists and describes all the statistical methods used to analysis the data; cite the statistical software used.

**Response:** Statistical analysis paragraph was included into the methods section. Line 100.

Minor revisions:
2. Abstract: Replace “neither” with “either.”

**Response:** Modified accordingly. Line 31.

3. Provide the percentage that corresponds to 16/57.

**Response:** Modified accordingly. Line 109.

4. Table 2: Provide corresponding percentages for categorical factors. For instance, for sex provide the percentage corresponding to 44/(44+13) Female & 13/(44+13) Male, and 7488/(7488+1961) Female & 1961/(7488+1961) Male.

**Response:** Modified accordingly. Table 2.

5. To assist in the review process, please add page and line numbers to the document.

**Response:** Added accordingly.

Reviewer #2:

This study identified the proportion of potentially infectious, nonsymptomatic HCWs which are either pre-symptomatic, asymptomatic or post-symptomatic. This topic is highlighted by WHO which mention the importance of this decisive factor on the spread of the pandemic. The authors tested an impressive number of HCWs from Karolinska University hospital, using methods described in detail in references 7 and 8: serology and RT PCR. Previously published studies (references 6, 9 and 10) by the same team of researchers, support the authors' interest in the real-time pandemic situation in Stockholm. The results of this study that the younger HCWs are potentially more infectious, based on real laboratory data, and the conclusion that they should get vaccinated, could be used like a model for other EU countries, to motivate their HCWs and general population to vaccinate themselves.

**Response:** We thank the reviewer for this important comment. We also believe that these results should motivate intervention policies for vaccinating the healthcare personnel at all ages in a systematic fashion.
